# Estimation of resting metabolic rate in professional soccer players: A cross-sectional study comparing traditional predictive equations and a preliminary machine learning model against indirect calorimetry

**DOI:** 10.1371/journal.pone.0354973

**Published:** 2026-08-12

**Authors:** Carlos Abraham Herrera-Amante, Rodrigo Yáñez-Sepúlveda, Eduardo Báez-San Martín, César Octavio Ramos-García, Eduardo Guzmán-Muñoz, Rodrigo Olivares, José Francisco López-Gil

**Affiliations:** 1 Division of Health Sciences, Nutritional Assessment and Nutritional Care Laboratory, Tonalá University Center, University of Guadalajara, Tonalá, México; 2 Ibero-American Network of Researchers in Applied Anthropometry, Almería, Spain; 3 Faculty of Education and Social Sciences, Universidad Andres Bello, Viña del Mar, Chile; 4 Laboratorio de Fisiología del Ejercicio y Rendimiento Deportivo, Facultad de Ciencias de la Actividad Física y del Deporte, Universidad de Playa Ancha, Valparaíso, Chile; 5 Facultad de Ciencias de la Vida, Carrera de Entrenador Deportivo, Universidad Viña del Mar, Viña del Mar, Chile; 6 Facultad de Salud, Escuela de Kinesiología, Universidad Santo Tomás, Talca, Chile; 7 Facultad de Educación, Escuela de Pedagogía en Educación Física, Universidad Autónoma de Chile, Talca, Chile; 8 Escuela de Ingeniería Informática, Universidad de Valparaíso, Valparaíso, Chile; 9 School of Medicine, Universidad Espíritu Santo, Samborondón, Ecuador; 10 Vicerrectoría de Investigación y Postgrado, Universidad de Los Lagos, Osorno; Universiti Malaya, MALAYSIA

## Abstract

**Background:**

Resting metabolic rate (RMR) is a major component of total daily energy expenditure and varies according to age, sex, and body composition. Although indirect calorimetry (IC) is the gold standard, predictive equations are widely used in practice. This study evaluated the agreement between twelve traditional RMR equations and IC in professional soccer players and explored a preliminary machine learning approach.

**Methods:**

Forty male professional soccer players (22.5 ± 4.4 years) were assessed. RMR measured by IC was compared with twelve predictive equations. A support vector regression (SVR) model was developed using anthropometric variables and evaluated under internal validation.

**Results:**

All equations showed poor concordance with IC (intraclass correlation coefficient [ICC]: −0.094 to 0.030) and overestimated RMR (8.38% to 36.38%). The SVR model achieved a mean absolute error of 169.3 kcal·day^-1^ and root mean square error (RMSE) of 190.7 kcal·day^-1^. Its prediction error was lower than the RMSE and average bias of traditional equations, indicating improved individual-level accuracy. However, it explained a limited proportion of variance (*R*^2^ = 0.169).

**Conclusion:**

Traditional equations showed poor agreement with indirect calorimetry in this sample of soccer players. These findings highlight the risks of relying on conventional predictive equations in professional athletes. Preliminary results suggest that machine learning models may improve estimation under internal validation, providing a proof of concept for data-driven approaches in this field. However, their predictive capacity remains limited, and external validation in larger and independent cohorts is required.

## Introduction

Soccer is the most popular team sport worldwide. The game requires the continuous interaction of technical and tactical skills combined with substantial physical effort due to its intermittent nature. It involves repeated bouts of high-intensity, multidirectional actions, such as sprints, changes of direction, jumps, and tackles, interspersed with periods of low-intensity activity and recovery, imposing a considerable physiological and metabolic load on players [1–3]. During a match, soccer players typically cover between 9 and 14 km, expending approximately 5,700 kJ (1,360 kcal), of which 70–90% is supplied by oxidative metabolism, with muscle glycogen serving as the primary substrate [1–4]. Recent evidence also shows a marked increase in high-intensity actions, with central and wide midfielders and full-backs covering greater total and high-speed distances than forwards or central defenders [2–6]. Consequently, individualized nutritional planning is essential to adequately meet the demands of training and competition [5]. In this context, accurate estimation of energy requirements becomes a key challenge in applied sports nutrition.

Accurate assessment of resting metabolic rate (RMR) is fundamental for determining athletes’ energy requirements, as it represents approximately 40–70% of total daily energy expenditure (TDEE) [7]. Although this proportion varies with training load and frequency, RMR remains one of the most influential components of energy metabolism. Precise measurement allows caloric intake to be adjusted to physiological demands, supporting performance, recovery, and the prevention of energy imbalance [8].

Indirect calorimetry (IC) is widely used as the gold-standard method for determining RMR because it estimates energy expenditure from oxygen consumption (VO_2_) and carbon dioxide production (VCO_2_), applying the Weir equation to quantify metabolic energy expenditure [9]. It also enables the determination of the respiratory exchange ratio (RER) and substrate utilization. However, its application requires specialized equipment, trained personnel, and controlled testing conditions, such as fasting, rest, and temperature control, which limit its routine use in applied sports settings [8,10].

Given these constraints, practitioners and researchers frequently rely on predictive equations to estimate RMR from simple variables such as body mass, stature, age, or body composition. The Harris–Benedict and Cunningham equations are among the most commonly used and are recommended by the American College of Sports Medicine (ACSM) for athletic populations [11]. However, an important limitation is that most of these equations were originally developed in non-athletic or heterogeneous populations, which may compromise their validity in highly trained athletes.

Nonetheless, recent evidence shows inconsistent performance in athletes. In their 2023 systematic review and meta-analysis, O’Neill et al. reported that although both equations demonstrated acceptable mean accuracy relative to IC, they showed very high heterogeneity (I^2^ ≈ 80–93%) across studies [8]. The Cunningham equation, particularly its fat-free mass (FFM) based version, tended to provide more accurate group-level estimates, whereas the Harris–Benedict equation frequently underestimated RMR in highly trained athletes and occasionally overestimated it in recreationally active individuals. Importantly, both equations demonstrated limited precision at the individual level and substantial variability influenced by sex, body composition, and competitive level, which restricts their practical applicability.

Several alternative equations have been proposed, but their validity varies widely across populations. A recent study in youth Premier League players developed a soccer-specific equation based on FFM, improving accuracy relative to traditional models [12]. However, the sample consisted primarily of adolescent players, limiting generalization to adult professionals whose physiological maturity, muscle mass, and metabolic demands differ substantially.

Despite methodological developments, research on RMR estimation in adult professional soccer players remains scarce, and most available equations were developed in general or youth populations, reducing their applicability in high-performance contexts. This limitation highlights the need for more accurate, individualized, and potentially data-driven approaches to estimate RMR in elite athletes.

Therefore, this study aimed to: a) evaluate the level of agreement between several traditional RMR predictive equations and indirect calorimetry in professional soccer players; and b) pilot the development of multiple machine learning–based estimation models by comparing their relative error against indirect calorimetry. By focusing on the limitations of existing equations, this study also explores whether machine learning approaches may provide a preliminary alternative for improving individual-level prediction.

## Materials and methods

### Study design

This was a descriptive, cross-sectional study. Participants were directed to the testing location for data collection only once. Guidelines for reporting observational studies (Strengthening Reporting of Observational Studies in Epidemiology [STROBE]) were followed [13].

### Setting

The present study was conducted during the Opening Tournament 2014. All assessments were carried out in Guadalajara, Jalisco, Mexico, from October 1–7, 2014, with the main venue being the Atlas Clubhouse. This study was approved by the Biosecurity, Research and Ethics Committees of the Division of Health Sciences of the University of Guadalajara, University Center of Tonalá (Code: CEI-062020–01). Individual information (descriptive data, player position, and experience) was extracted after completing a brief practical form for sports nutrition counseling [14]. All participants signed an informed consent form after being fully and correctly informed by the principal investigator of the participation requirements, purpose, risks, and benefits of the study. Signed parental consent was obtained for participants aged < 18 years. This study was conducted in accordance with the ethical principles for medical research of the International Guidelines for Good Clinical Practice and the Declaration of Helsinki [15]. The data were originally collected in 2014 within the framework of routine sports-nutrition assessment; ethical approval for the retrospective analysis and for the publication of these anonymized data was subsequently granted by the committee cited above (Code: CEI-062020–01). The informed consent signed by the participants, and the parental consent obtained for those aged < 18 years, explicitly covered the use of the collected data for research purposes and their subsequent anonymized public sharing.

### Participants

A total of forty male professional soccer players (age: 22.5 ± 4.4 years; stature: 172.1 ± 8.6 cm; body mass: 64.7 ± 12.4 kg; competitive experience: 1.6 ± 4.9 years; weekly training volume: 15 ± 3.2 hours) participated in the study. All were actively competing in the national league. Participants were invited to the study if they met the following inclusion criteria: i) resided in the Atlas Clubhouse, and ii) attended the evaluation area prior to their competition. Exclusion criteria were: i) attending the evaluation area without meeting the required conditions, and ii) not providing written consent (or parental consent for participants under 18 years of age) for the procedures or the disclosure of data for research purposes at the time of evaluation.

### Data sources and measurements

Participants attended the designated testing area after an overnight fast of 7–8 hours and at least 12 hours after their last exercise session. They were instructed to refrain from consuming alcohol, stimulants, food, or dietary supplements (including coffee, tea, chocolate, carbonated beverages, and energy drinks) for at least 48 hours prior to the beginning of the study.

### Resting metabolic rate

RMR was measured using indirect calorimetry (Breezing®, Arizona, USA), which was calibrated prior to each test and has been previously validated by Xian et al. [16]. Before the measurements, participants completed a 5–10-minute familiarization session with the calorimetry equipment. RMR was calculated from minute-by-minute oxygen consumption (VO_2_, mL·min^-1^) and carbon dioxide production (VCO_2_, mL·min^-1^) using the Weir equation [17], while participants remained in the supine position for an average of 12–15 minutes. All assessments were conducted following the methods recommended by the Academy of Nutrition and Dietetics for RMR measurement in adults [18]. Measurements were performed in the morning in a quiet, thermoneutral room with ambient temperature maintained between 18 and 22 °C and relative humidity between 30 and 40%, monitored using a thermal stress meter (EXTECH® HT30). Steady state was defined following the Academy of Nutrition and Dietetics best-practice criteria [18]. As the Breezing® system uses a single-use sensor with a fixed measurement duration of approximately 5 minutes, participants completed the familiarization period before testing to ensure stable breathing patterns and compliance with testing procedures. Respiratory exchange ratio (RER) values were monitored to ensure they remained within the physiologically plausible range (0.7–1.0). When RER values, testing conditions, or participant preparation did not comply with the standardized protocol requirements, the assessment was rescheduled and repeated on a different day. Consequently, no participants were excluded from the final analysis on this basis.

### Estimation of basal and resting metabolic rate

This study included twelve traditional predictive equations, six of which estimate basal metabolic rate (BMR) and the other six estimate RMR. Given this critical distinction, and to compare and analyze these estimates with RMR measured by indirect calorimetry, BMR estimates were adjusted by adding 10% to obtain an estimated RMR. This correction was applied primarily to account for differences in post-absorptive conditions and the energetic cost of arousal between BMR and RMR. Therefore, all twelve estimation equations were analyzed based on either the measured RMR or the calculated RMR. This same strategy has been employed previously in other studies [7].

### Anthropometric measurements

The anthropometric measurements were performed according to the protocols of the International Society for the Advancement of Kinanthropometry (ISAK) by a Level 3 certified anthropometrist. Body mass (kg) was determined by using a digital scale with a precision of 50 g (SECA® 874, Hamburg, Germany). To assess stretch stature (cm), a stadiometer with an accuracy of 1 mm (SECA® 217, Hamburg, Germany) was used.

### Study sample

Non-probabilistic convenience sampling was used because of the difficulty in obtaining large samples of professional soccer players.

### Statistical methods

Normality was assessed using the Shapiro–Wilk test, which confirmed a normal distribution of the variables (*p* > 0.05). The sample size of 40 professional athletes provided a bias precision of approximately ±115 kcal·day^-1^ (95% CI) and adequate statistical power (>80%) to detect a minimum concordance of *ρ* ≥ 0.78 against a reference threshold of *ρ* = 0.50, a value commonly considered acceptable in metabolic validation studies involving elite athletic populations.

Predictive RMR was estimated using twelve traditional predictive equations: Cunningham (1980) [19], FAO/WHO (1985) [20], Harris and Benedict (1918) [21], Henry (2005) [22], Valencia et al. (1994) [23], Wong et al. (2012) [24], De Lorenzo et al. (1999) [25], Hannon et al. (2020) [12], Kim et al. (2015) [26], Mifflin et al. (1990) [27], Müller et al. (2004) [28], and Owen et al. (1987) [29]. Each equation was applied following its original formulation, using directly measured anthropometric variables such as body mass, stretch stature, age, sex, and lean mass when required.

All analyses were performed in Python 3.11 using the pandas, pingouin, numpy, and matplotlib libraries. Agreement between measured and estimated RMR values was evaluated through a multimethod concordance framework. This included the calculation of the intraclass correlation coefficient (ICC) using a two-way mixed-effects model (absolute agreement), interpreted according to the criteria proposed by Koo and Li [30]. Lin’s concordance correlation coefficient (CCC) was computed to jointly assess precision and accuracy, while Pearson’s correlation coefficient (r) and its associated *ρ*-value quantified the linear association between methods. Absolute and systematic discrepancies were determined using the root mean square error (RMSE) and mean bias, respectively. Individual-level agreement was assessed through Bland–Altman analysis, including the estimation of the mean difference and the 95% limits of agreement (LoA = mean ± 1.96 × SD). An RMSE < 200 kcal·day^-1^ and an absolute bias < 10% of the measured RMR were considered thresholds of clinically acceptable agreement.

To evaluate the magnitude of the discrepancies between predicted and measured RMR, Hedges' g g effect size was calculated. This statistic allowed the standardized difference between methods to be quantified, accounting for within-sample variability. Values near zero reflected a high degree of agreement, whereas positive values indicated systematic overestimation by predictive equations. Interpretation followed Cohen’s (1988) criteria adapted for Hedges' g, considering 0.2 as a small effect, 0.5 as a medium effect, and 0.8 or greater as a large effect [31].

Finally, an additional comparison between each predictive model and indirect calorimetry (IC) was performed using the relative error (RE), calculated as:


$$\begin{array}{c} R_{E}=\frac{\left[EE^{est}-EE^{IC}\right]}{EE^{IC}}\times \text{1}\text{0}\text{0}, \end{array}$$ (1)

where *EE*^*est*^ corresponds to the estimated RMR and *EE*^*IC*^ to the measured value obtained through IC. This metric quantified the percentage deviation of each equation from the reference method, providing a complementary indicator of model performance at the individual level.

### Machine learning analysis

#### Study design and population.

A predictive modeling analysis was conducted using the dataset of 40 professional soccer players described previously. The dataset included directly measured anthropometric variables, body mass and stature, and derived indicators such as body mass index (BMI) and body surface area (BSA). All measurements were collected under standardized conditions following the procedures outlined in earlier sections.

#### Data preprocessing.

A structured preprocessing pipeline was applied to ensure data quality and enhance model robustness. To prevent data leakage, the dataset was first partitioned into a training subset and a held-out internal-validation subset (see below), and all subsequent preprocessing steps were fitted exclusively on the training data and then applied, unchanged, to the held-out subset. Potential outliers were screened using the inter-quartile range (IQR) criterion; this criterion did not flag any observation as an outlier, so that all 40 participants were retained, and the metrics reported below therefore correspond to the complete sample. The predictor variables were standardized using a RobustScaler transformation, which limits the influence of residual outliers while preserving the central tendency of the variables.

Additional feature engineering included the creation of nonlinear and interaction terms to capture complex relationships between anthropometric predictors. The full set of input features, including primary, derived, nonlinear, and interaction variables, is detailed in Table 1. Because the derived predictors (body mass index, body surface area, squared body mass, and the body mass × stature interaction) are mathematical functions of body mass and stature, the predictor set was collinear by construction; the variance inflation factors confirmed severe multicollinearity, with values for the derived terms far above the conventional threshold of 10 (approximately 3.5 × 10^5^ for body mass, 6.6 × 10^5^ for body surface area, 1.8 × 10^5^ for the body mass × stature interaction, 1.1 × 10^5^ for squared body mass, and 1.6 × 10^3^ for body mass index, versus 1.7 for age). For this reason and given the small sample size (*n* = 40), the models were used solely for prediction, and no inference is made regarding the relative importance or the independent contribution of individual predictors.

**Table 1:** Input features used in the machine learning models, including primary, derived, nonlinear, and interaction variables.

Category	Feature	Description / Formula	Justification
Primary	Body Mass (kg)	Direct measurement	Primary determinant of RMR
	Stretch stature (cm)	Direct measurement	Indicator of body size
	Age (years)	Recorded value	Influences metabolic rate decline
Derived	BMI (kg/m²)	Body mass / Stretch stature²	Mass-to-volume relationship
	BSA (m²)	DuBois & DuBois formula	Proxy for heat exchange surface
Nonlinear	Mass² (kg²)	Squared body mass	Captures nonlinear scaling of metabolism
Interaction	Mass × Stature	Product of body mass and stretch stature	Represents combined body size effect

Model development followed a single random partition of the dataset into a training subset (80%) and a held-out internal-validation subset (20%). Hyperparameters were tuned by 5-fold cross-validation applied only within the training subset, while the held-out subset was used once to estimate the performance metrics reported below. No separate leave-one-out cross-validation was used to obtain the final estimates. Because both hyperparameter tuning and performance estimation derived from a single small dataset (*n* = 40), and because no external validation set was available, the resulting metrics may be optimistically biased; they are therefore interpreted as exploratory (proof-of-concept) results rather than as confirmed estimates of out-of-sample accuracy.

#### Model evaluation framework.

A comprehensive evaluation framework was implemented to assess multiple methodological families of machine learning algorithms. A total of sixteen models were tested, including linear approaches (Ridge, Lasso, ElasticNet), support vector regression (SVR and optimized SVR), tree-based ensembles (Random Forest, Gradient Boosting, XGBoost, LightGBM), Bayesian algorithms (Bayesian Ridge, Gaussian Process), artificial neural networks (Multilayer Perceptron), and advanced ensemble strategies such as stacking and meta-model combinations.

All models were optimized through cross-validation and hyperparameter tuning. Predictive performance was evaluated using mean absolute error (MAE), root mean square error (RMSE), the coefficient of determination (*R*^2^), mean absolute percentage error (MAPE), and computational time.

#### Model selection and validation.

The optimized Support Vector Regression model (SVR_Optimized) showed the most favorable performance in the internal validation among the algorithms evaluated. Model validation included a detailed inspection of learning curves to determine generalization capacity and detect potential overfitting. Residual diagnostics were conducted to verify assumptions of normality, homoscedasticity, and independence. Robustness was further assessed by comparing the performance of the SVR_Optimized model with that of the ten best-performing alternatives across all evaluation metrics.

#### Hyperparameter optimization of the SVR model.

The Support Vector Regression (SVR) model was optimized using a grid search strategy (GridSearchCV) implemented in scikit-learn. A radial basis function (RBF) kernel was selected to capture nonlinear relationships between predictors and RMR. Hyperparameter tuning was performed using 5-fold cross-validation to enhance model robustness and reduce overfitting risk.

The following parameter ranges were explored:

C: [0.1, 1, 10, 100, 1000]
ε: [0.01, 0.1, 0.2, 0.5]
γ: [10^-3^, 10^-2^, 10^-1^, 1, ‘scale’, ‘auto’]

The optimal configuration selected was:

C = 10, ε = 0.1, γ = ‘scale’.

This configuration provided the best trade-off between bias and variance, achieving the lowest prediction error among all evaluated models.

#### Software and implementation.

All machine learning analyses were conducted in Python (version 3.11) using the scikit-learn, XGBoost, and LightGBM libraries. Computations were executed on a standard workstation (Intel Core i7 processor, 16 GB RAM), ensuring reproducibility and efficient performance. The entire modeling pipeline was version-controlled and documented to facilitate transparency and replicability.

## Results

Table 2 shows the comparison between the RMR measured by indirect calorimetry (Breezing®) and the values estimated using various predictive equations. The mean and standard deviation in kcal·day^-1^ are included, along with the relative percentage error with respect to the measured value. The results show that all equations overestimated RMR compared to indirect calorimetry, with relative errors ranging from 8.38% to 36.38%. The equation of Owen et al. (1987) [29] showed the lowest relative error, followed by those of Kim et al. (2015) [26], Mifflin et al. (1990) [27], and Müller et al. (2004) [28], whereas FAO/ WHO (1985) [20], Harris and Benedict (1918) [21], and Wong et al. (2012) [24] showed the largest discrepancies. These results suggest that equations developed from more recent or specific populations may not be adequately adjusted to the group evaluated, highlighting the importance of validating predictive equations according to the characteristics of the sample.

**Table 2:** Comparison of resting metabolic rate (RMR) values measured and estimated using different predictive equations.

Equation / Method	Mean (kcal·day^-1^)	SD (kcal·day^-1^)	Relative error (%)
Indirect Calorimetry (Breezing®)	1511.5	348.3	—
Cunningham (1980) [19]	1889.3	125.4	31.2
FAO/WHO (1985) [20]	1963.8	150.8	36.4
Harris & Benedict (1918) [21]	1912.4	160.8	32.8
Henry (2005) [22]	1893.3	156.9	31.5
Valencia et al. (1994) [23]	1802.5	129.7	25.2
Wong et al. (2012) [24]	1900.8	126.1	32.1
De Lorenzo et al. (1999) [25]	1773.9	160.3	23.2
Hannon et al. (2020) [12]	1887.2	83.7	31.2
Kim et al. (2015) [26]	1665.7	99.5	16.1
Mifflin et al. (1990) [27]	1672.9	124.2	16.2
Müller et al. (2004) [28]	1696.8	99.9	17.9
Owen et al. (1987) [29]	1559.2	89.9	8.4

Table 3 shows the results of the concordance analysis between RMR values measured by indirect calorimetry and those estimated by various predictive equations, using reliability and accuracy indicators. The intraclass correlation coefficients (ICC) and Lin's concordance coefficients (CCC) were very low in all equations (≤0.03), indicating poor reliability and concordance with the reference method. Likewise, Pearson's correlation coefficients (r) ranged from −0.205 to 0.080, without reaching statistical significance (*ρ* > 0.05 in all cases), which shows an absence of a significant linear relationship between the methods. In terms of errors, the RMSE values ranged from 355.82 to 581.95 kcal·day^-1^, and the average biases were positive in all equations (ranging from 48 to 452 kcal·day^-1^), indicating a systematic tendency to overestimate RMR compared to indirect calorimetry. The limits of agreement (LoA) of the Bland–Altman analysis showed wide margins of variability (up to approximately ±1100 kcal·day^-1^), reinforcing the low precision and high dispersion of the estimates. Overall, these results reflect that none of the predictive equations evaluated showed adequate agreement with the direct measurement of RMR. Within this overall poor agreement, the highest intraclass and concordance coefficients corresponded to Henry's equation (2005) [22] (ICC = CCC = 0.030), whereas the lowest bias and RMSE corresponded to Owen's equation (1987) [29] (bias = 47.8 kcal·day^-1^; RMSE = 355.82 kcal·day⁻¹); however, agreement remained poor for all equations.

**Table 3:** Evaluation of the concordance and accuracy of predictive equations compared to indirect calorimetry for estimating resting metabolic rate (RMR).

Predictive Equation	ICC	CCC	RMSE	Bias	SD diff	LoA low	LoA high
			(kcal·day^-1^)	(kcal·day^-1^)			
**Henry (2005)**	0.030	0.030	528.6	381.8	370.3	−343.9	1107.5
**Müller et al. (2004)**	0.021	0.021	398.7	185.3	357.5	−515.4	886.0
**FAO/WHO (1985)**	0.019	0.019	581.9	452.3	370.8	−274.4	1179.1
**Hannon et al. (2020)**	0.006	0.006	514.6	376.1	355.7	−321.2	1073.4
**Mifflin et al. (1990)**	0.015	0.015	396.3	161.5	366.5	−556.9	879.8
**Owen et al. (1987)**	0.015	0.014	355.8	47.8	357.0	−652.1	747.6
**Cunningham (1980)**	0.013	0.013	522.4	377.8	365.3	−338.2	1093.9
**Valencia et al. (1994)**	0.012	0.012	465.5	291.0	367.9	−430.1	1012.3
**Harris & Benedict (1918)**	0.011	0.011	548.4	400.9	379.0	−342.0	1143.8
**De Lorenzo et al. (1999)**	0.010	0.010	458.3	262.4	380.5	−483.4	1008.4
**Wong et al. (2012)**	0.009	0.009	531.7	389.3	366.8	−329.6	1108.3
**Kim et al. (2015)**	−0.094	−0.091	406.8	154.2	381.2	−593.0	901.5

This pattern was consistent across all evaluated equations. Agreement remained poor, with ICC and CCC values close to zero or negative, indicating a minimal ability to reproduce the measured values. The intraclass correlation coefficients (ICC) ranged from −0.094 to 0.030, denoting a lack of consistency between methods and unacceptable systematic variability according to international methodological standards [30]. Similarly, Lin's concordance coefficients (CCC) remained low (−0.091 to 0.030), reflecting poor simultaneous precision and accuracy. Pearson's correlation coefficients (*r* = −0.205 to 0.080; *ρ* > 0.05) also confirmed the absence of a significant linear association between predicted and measured values. The RMSE values remained high (355.82 to 581.95 kcal·day^-1^), and the bias indicated systematic overestimation in most equations, reaching up to +452 kcal·day^-1^. The wide limits of agreement (up to ±1100 kcal·day^-1^) further illustrate the substantial individual variability. These findings indicate that traditional equations perform inadequately for estimating resting metabolism at the individual level.

**Fig 1:**
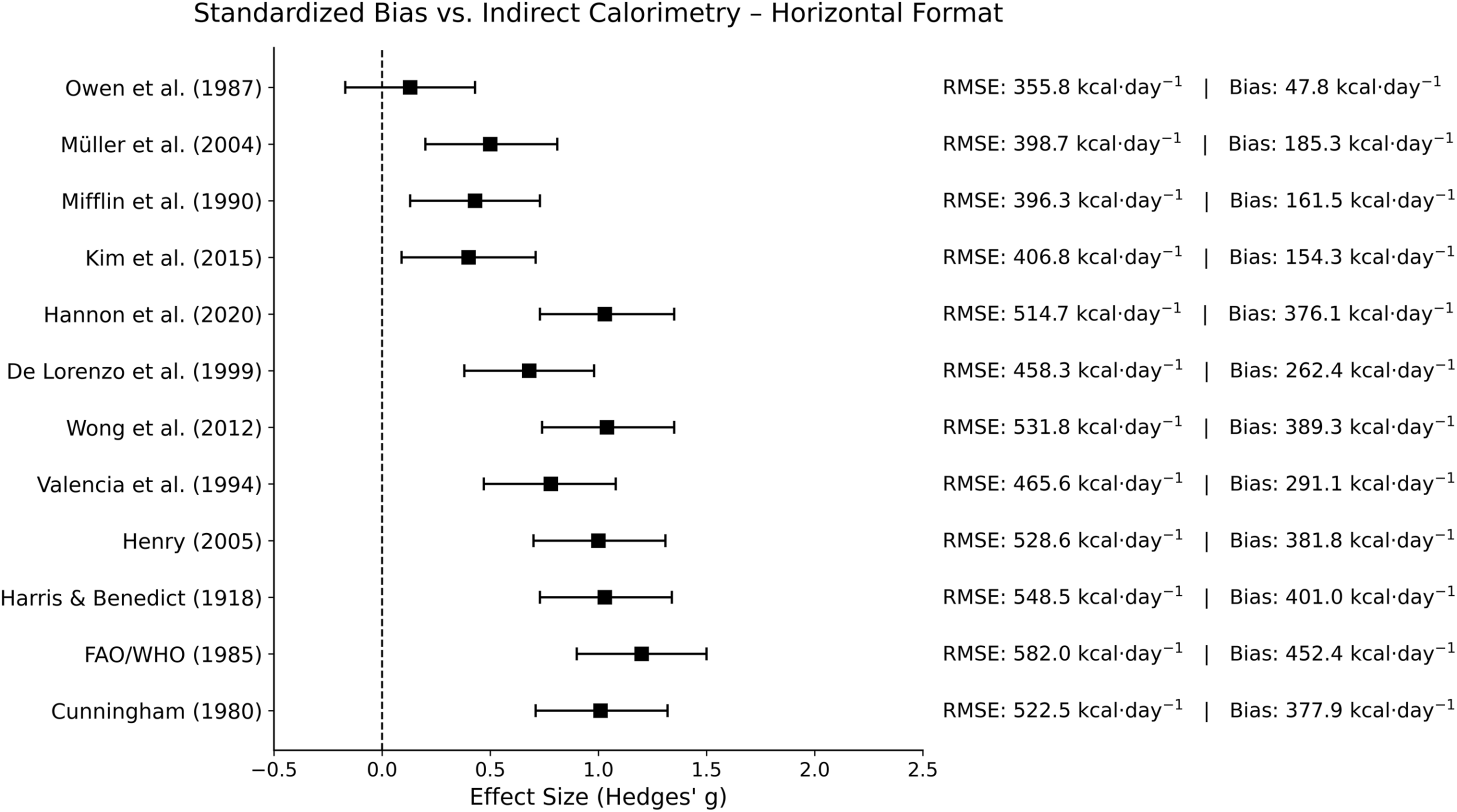
Effect size (Hedges' g) of predictive equations compared with indirect calorimetry for estimating resting metabolic rate (RMR).

In Fig 1, a forest plot illustrates the effect sizes (Hedges’ g) comparing several predictive equations for RMR. Each horizontal line represents the 95% confidence interval for the corresponding equation, with the central square denoting the estimated effect size. The figure shows that the equations proposed by FAO/ WHO (1985), Wong et al. (2012), Harris & Benedict (1918), Cunningham (1980), and Henry (2005) display the largest effect sizes, indicating greater deviations from the reference standard. In contrast, the equations of Owen et al. (1987), Kim et al. (2015), and Mifflin et al. (1990) present the smallest effect sizes, suggesting comparatively better agreement.

To provide a more comprehensive and transparent interpretation of model performance, RMSE and bias values for the main predictive equations and the optimized SVR model are also presented in Table 4.

**Table 4:** Effect size, RMSE, and bias of predictive equations and the optimized SVR model compared to indirect calorimetry.

Method / Equation	Hedges’ g [95% CI]	Effect Size Classification	RMSE (kcal·day^-1^)	Bias (kcal·day^-1^)
SVR Optimized (ML)	−0.05 [−0.36, 0.26]	Small	190.7	0.0
Owen et al. (1987)	0.17 [−0.27, 0.61]	Small	355.8	47.8
Müller et al. (2004)	0.67 [0.21, 1.13]	Medium	398.7	185.3
Mifflin et al. (1990)	0.62 [0.16, 1.08]	Medium	396.3	161.5
Kim et al. (2015)	0.60 [0.14, 1.06]	Medium	406.8	154.2
Hannon et al. (2020)	1.25 [0.76, 1.74]	Large	514.7	376.1
De Lorenzo et al. (1999)	0.90 [0.43, 1.37]	Large	458.3	262.4
Wong et al. (2012)	1.35 [0.85, 1.85]	Large	531.8	389.3
Valencia et al. (1994)	1.05 [0.57, 1.53]	Large	465.6	291.1
Henry (2005)	1.28 [0.79, 1.77]	Large	528.6	381.8
Harris & Benedict (1918)	1.34 [0.84, 1.84]	Large	548.4	400.9
FAO/WHO (1985)	1.55 [1.03, 2.07]	Large	581.9	452.4
Cunningham (1980)	1.31 [0.81, 1.81]	Large	522.5	377.9

Abbreviations: RMSE, root mean square error; CI, confidence interval; ML, machine learning.

Table 5 shows that the optimized Support Vector Regression (SVR) model showed the most favorable internal-validation results for predicting energy expenditure estimated by indirect calorimetry in professional soccer players. It had a mean absolute error (MAE) of 169.3 kcal·day^-1^, a root mean square error (RMSE) of 190.7 kcal·day^-1^, and a mean absolute percentage error (MAPE) of 11.4%, with a coefficient of determination (*R*^2^) of 0.169. These values indicate a lower internal-validation error than that obtained with the traditional equations; nevertheless, this coefficient of determination shows that most of the between-individual variability in RMR remained unexplained. The model was also computationally efficient (1.2 s), with 95% coverage; however, the small sample size (*n* = 40) and the absence of external validation preclude any firm conclusion regarding its stability or generalization. Compared with the other algorithms evaluated, including ensemble methods and neural networks, the optimized SVR model achieved the most favorable trade-off between error, robustness, and processing time; nevertheless, these results should be regarded as exploratory (proof-of-concept), and external validation in larger, independent cohorts is required before any practical application can be considered.

**Table 5:** Performance metrics of predictive models for indirect calorimetry estimation in professional soccer players.

Model	MAE (kcal·day^-1^)	RMSE (kcal·day^-1^)	*R* ^2^	MAPE	Time
SVR Optimized	169.3	190.7	0.169	11.40%	1.2s
LightGBM	196.2	210.8	−0.013	12.90%	0.7s
ElasticNet CV	196.2	210.8	−0.013	12.90%	1.1s
Bayesian Ridge	196.3	210.8	−0.013	12.90%	0.0s
SVR	196.7	211.9	−0.024	12.90%	0.0s
Stacking Ensemble	202.0	229.6	−0.203	12.70%	2.5s
Elastic Net	227.9	249.9	−0.425	15.00%	0.0s
Ridge	258.8	294.9	−0.985	17.10%	0.0s
KNN	262.0	286.8	−0.878	18.00%	1.7s
Lasso	273.2	321.3	−13.561	18.20%	0.0s
XGBoost	352.2	389.2	−24.579	24.00%	3.0s
Gradient Boosting	354.9	397.3	−26.027	24.30%	1.8s
Decision Tree	563.8	638.5	−83.068	39.20%	0.0s
Linear Regression	655.3	902.4	−175.873	42.60%	0.0s
Gaussian Process	924.8	1100.8	−266.57	60.80%	0.2s
MLP	1110.2	1146.1	−289.821	72.70%	0.7s

Abbreviations: MAE, mean absolute error; RMSE, root mean square error; MAPE, mean absolute percentage error; SVR, support vector regression; GBM, gradient boosting machine; CV, cross-validation; KNN, k-nearest neighbors; MLP, multilayer perceptron.

**Fig 2:**
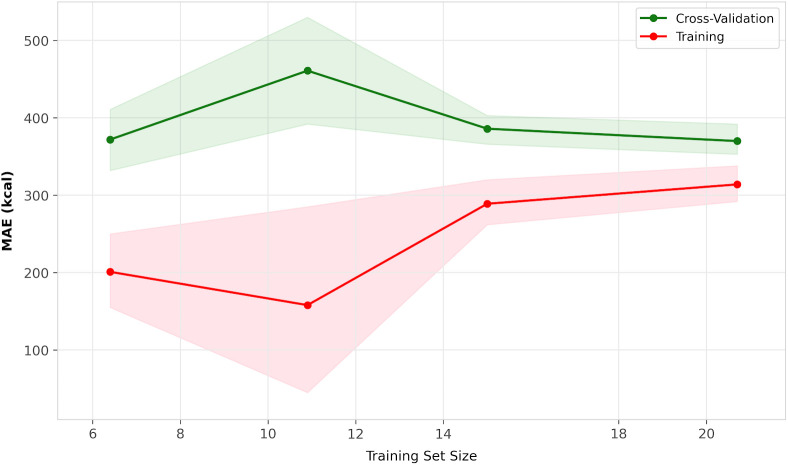
Learning curve of the optimized support vector regression (SVR) model.

Fig 2 shows the learning curve of an optimized SVR model, illustrating the mean absolute error (MAE) in kilocalories for both the training (red) and cross-validation (green) sets as the training set size increases. The shaded areas represent variability or confidence intervals. The curve indicates a decrease in error with larger sample sizes, suggesting that the model’s performance stabilizes as more data become available.

**Fig 3:**
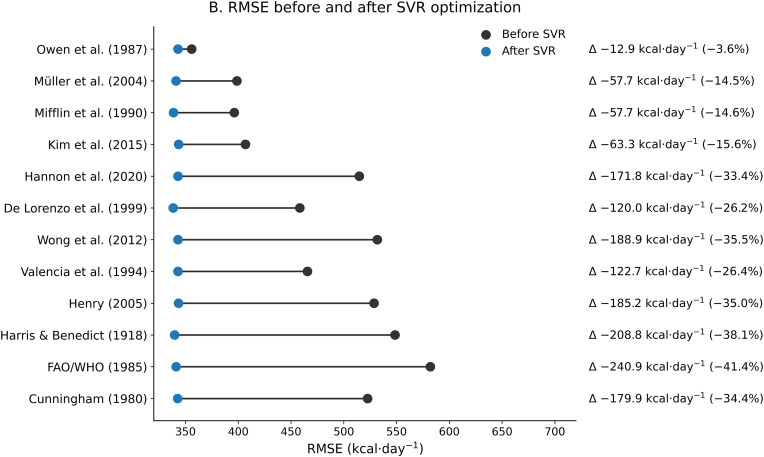
RMSE before and after SVR optimization.

Fig 3 compares the RMSE of each equation before and after adjustment with SVR. The gray dots represent the original error, and the blue dots represent the adjusted error, joined by black lines that indicate the magnitude of the change. A consistent reduction in RMSE is observed in all equations, with absolute and relative improvements noted on the right. This pattern indicates that, in this internal-validation analysis, the SVR-adjusted estimates were associated with a lower RMSE than the traditional equations; given the small sample and the absence of external validation, this observation should be interpreted as exploratory.

## Discussion

### Key findings

The main finding of this study is that commonly used RMR prediction equations show poor agreement with indirect calorimetry (IC) in professional male soccer players. All evaluated equations displayed very low intraclass correlation coefficients, approaching zero, and consistently overestimated measured RMR. The magnitude of overestimation ranged from 47.8 to 452.3 kcal·day^-1^, representing relative errors between 8.38% and 36.38%. None of the equations achieved the predefined acceptable agreement with IC. The equation with the highest concordance in our dataset was the Henry (2005) equation, although reliability remained poor (ICC = 0.030; CCC = 0.030). The equation with the smallest bias and RMSE, Owen (1987), was nevertheless highly imprecise, displaying wide limits of agreement (approximately 1,400 kcal·day^-1^), which were comparable to those of the other equations. These findings indicate that none of the evaluated equations are suitable for accurate individual-level estimation of RMR in this population. These findings align with recent results in Olympic athletes, where even the closest equation (Harris–Benedict) showed only moderate accuracy (mean error of –9 kcal·day^-1^, ICC 0.52) and all formulas proved inadequate for precise individual assessment [32]. Our results reinforce this concern within the context of intermittent–endurance sports such as professional soccer.

### Interpretation

The low performance of traditional RMR equations in this sample can be largely explained by population mismatch. Each equation was developed from a specific demographic or physiological reference group that differs markedly from highly trained athletes. As a result, equations calibrated on general populations often fail to capture the metabolic adaptations associated with chronic high-intensity training, elevated lean mass, and sport-specific physiological demands.

The discrepancies observed across equations illustrate this issue clearly. The Harris–Benedict equation (1918) [21], although shown to perform adequately in other male athletic cohorts [33,34], substantially overestimated RMR in our players by an average of 400.9 kcal·day^-1^ (32.8%). The wide limits of agreement in our data indicate that the Harris–Benedict formula is unsuitable for individual predictions in soccer players. This inconsistency likely reflects fundamental differences between early 20th-century adults from whom the equation was derived and modern professional athletes.

Similarly, the Mifflin–St Jeor equation (1990) [27], originally developed in overweight, sedentary adults, typically underestimates energy needs in athletic populations. In our sample, however, the equation produced a smaller bias (161.5 kcal·day^-1^) but remained unreliable, as its root-mean-square error (396.3 kcal·day^-1^) was still unacceptably large. The Cunningham equation (1980) [19], which incorporates fat-free mass (FFM) and is often recommended for athletes, also performed poorly by overestimating RMR by 377.8 kcal·day^-1^ (31.2%). This may stem from inaccuracies in FFM estimation or differences between our athletes and the group that informed the original Cunningham model.

The Owen equation (1987) [29] produced the lowest mean error (47.8 kcal·day^-1^), but this apparent accuracy was misleading because its limits of agreement were wide, spanning nearly 1,400 kcal·day^-1^, comparable to those of the other equations. Thus, despite showing a near-zero average bias at the group level, Owen’s equation was highly imprecise and unreliable for individual estimations. Meta-analytic evidence supports this interpretation, as several analyses have identified Owen’s equation as one of the least accurate for athletes.

Collectively, these findings indicate that equations relying solely on body mass, stretch stature, age and sex implicitly assume normative body composition profiles that do not reflect the unique anthropometric characteristics of professional soccer players. Although FFM is a strong determinant of RMR, as shown in studies such as Hannon (2020) [12] and Cunningham (1980) [19], our results reveal that even equations centered on FFM fail to generalize effectively to this athletic population. This underscores the need for sport-specific or data-driven models that better reflect the metabolic profiles of professional players.

In contrast with traditional predictive equations, the machine-learning model demonstrated improved predictive accuracy under internal validation procedures. The optimized SVR model achieved an MAE of 169.3 kcal·day^-1^, an RMSE of 190.7 kcal·day^-1^, and a MAPE of 11.4%, while explaining a modest proportion of the variance (*R*^2^ = 0.169). While this represents an improvement compared to traditional equations, the relatively low *R*^2^ indicates that a substantial proportion of the variability in RMR remains unexplained. This highlights the inherent complexity of metabolic processes and suggests that additional physiological and contextual variables (e.g., training load, hormonal status, and direct body composition measures) may be necessary to improve predictive performance.

Importantly, although the SVR model did not show clear signs of overfitting based on internal validation procedures and learning curves, these results should be interpreted with caution. The model should be considered exploratory, and its performance requires confirmation through external validation in independent and larger cohorts before any practical application can be recommended.

### Limitations

This study has several limitations that should be considered when interpreting the findings. First, the sample size was relatively small (*n* = 40), which limits statistical power and the ability of both traditional and machine-learning models to generalize beyond the studied sample. In addition, the dataset was collected in 2014, and therefore may not fully reflect the current physiological, training, and nutritional characteristics of professional soccer players, which have likely evolved substantially over the past decade. These factors represent a critical limitation for the generalizability and contemporary relevance of the findings.

Furthermore, the sample was drawn from a single professional club, which restricts variability in training adaptations, anthropometric characteristics, and metabolic profiles. This relative homogeneity may limit the applicability of the results to broader populations of soccer players.

Second, the absence of external validation represents a major limitation for the machine-learning model. Although the SVR demonstrated promising performance under internal validation, its generalizability to independent datasets remains unknown. External validation in multicenter cohorts is essential before considering its application in practice.

Third, many participants had not yet attained adult musculoskeletal maturity or adult body composition characteristics. This includes peak lean mass accumulation, a key determinant of RMR [12,35]. It is plausible that, upon reaching full maturity, agreement between RMR prediction equations and indirect calorimetry will improve, given that most models were derived from adults with stable body composition.

Fourth, although the Cunningham equation was implemented using fat-free mass (FFM) estimated per the original method (from body mass and age), body composition was not directly assessed. Direct measurement of FFM and related compartments (e.g., via dual-energy X-ray absorptiometry or bioelectrical impedance analysis) would likely yield more precise insight into RMR determinants in this cohort; the absence of such measurements limits our ability to fully explain inter-individual variability.

Finally, although RMR was measured under standardized conditions, inherent variability in pre-test diet, hydration, sleep, and circadian cycles could have modestly influenced indirect calorimetry measurements, as widely documented in RMR research [18].

These limitations underscore the need for cautious interpretation and highlight the importance of conducting validation studies across heterogeneous athletic cohorts, ideally incorporating longitudinal assessments to account for maturation and direct body composition measurements.

### Interpretation in context of evidence

When situated within the broader research landscape, our findings align with consistent evidence indicating that generic RMR prediction equations are unreliable for athletes. Numerous investigations in Olympic competitors, endurance athletes and mixed collegiate teams have demonstrated large individual errors and frequent over- or underestimation of true metabolic needs. These inaccuracies are generally attributed to differences in lean mass, organ size, metabolic efficiency and physiological adaptations that are not captured by equations derived from non-athlete samples.

The present results extend this pattern to professional soccer players, a group with unique demands involving intermittent high-intensity efforts and specific anthropometric and metabolic characteristics. The fact that all equations, including those typically recommended for athletes, display wide limits of agreement reinforces the broader consensus that traditional formulas lack precision for individual athletes, even when group means appear acceptable.

The exploratory findings of the machine-learning model are consistent with emerging applications of data-driven approaches in sports science. However, given the methodological limitations of the present study, these results should be interpreted as preliminary evidence rather than definitive support for implementation. Future studies incorporating larger, multicenter datasets and more comprehensive physiological variables are needed to confirm these observations.

### Generalizability

The generalizability of the findings is limited by the relatively small and homogeneous nature of the sample, which consisted exclusively of young male professionals from a single first-division soccer club. Therefore, these results cannot be assumed to apply to female players, youth athletes, amateur or semi-professional players, or athletes from other sports with different physiological demands. Similarly, cultural, ethnic and metabolic differences across geographic contexts may limit applicability beyond the population studied.

Nonetheless, despite these constraints, the central conclusion, that traditional RMR equations perform poorly in athletes, echoes a recurring pattern across the literature, suggesting that the issue is universal rather than population specific. In contrast, the machine-learning model should be considered a preliminary, proof-of-concept approach. Its application in real-world settings requires rigorous external validation across diverse populations, including different competitive levels, age groups, and geographic regions.

## Conclusions

Traditional equations for estimating RMR exhibit substantial inaccuracies when applied to athletic populations, as they were developed in non-sporting contexts and do not account for the specific physiological characteristics of soccer players. In this study, traditional equations overestimated RMR by 48–452 kcal·day^-1^, with an average bias of approximately 290 kcal·day^-1^. These findings highlight the risks of relying on conventional predictive equations for estimating energy requirements in professional athletes. These discrepancies highlight the limited external validity of classical prediction models for professional soccer players.

The implementation of machine learning models, particularly the optimized SVR, showed improved predictive performance under internal validation conditions. The model achieved a mean absolute error of 169.3 kcal·day^-1^ and an RMSE of 190.7 kcal·day^-1^. Importantly, its prediction error (MAE) was lower than the RMSE and average bias observed in traditional predictive equations, suggesting improved accuracy at the individual level. However, the relatively low coefficient of determination (*R*^2^ = 0.169) indicates that a substantial proportion of the variability in RMR remains unexplained.

Overall, these findings should be considered preliminary and exploratory. The present results provide a proof of concept supporting the potential of data-driven approaches for improving RMR estimation in sport-specific contexts. While machine learning approaches may contribute to improving predictive models, their application in practice requires cautious interpretation and rigorous external validation in larger and more diverse populations.

Future research should focus on incorporating additional physiological and contextual variables and validating these models in multicenter cohorts to enhance their robustness and generalizability.
