# Supplementary material for: Estimation of resting metabolic rate in professional soccer players: A cross-sectional study comparing traditional predictive equations and a preliminary machine learning model against indirect calorimetry
Source: PLoS One. 2026 Aug 12;21(8):e0354973. doi: 10.1371/journal.pone.0354973 (PMC13465844; doi:10.1371/journal.pone.0354973)
Supplement: Supplementary file 1 [file pone.0354973.s003.docx]

**Responses to Reviewer #1:**

1. The machine learning sample consisted of 40 cases, which may be insufficient for constructing a robust predictive model (as acknowledged by the authors in the limitations section). A small sample size increases the risk of overfitting. Although the study conducted cross-validation and learning curve analyses, if feasible, it would be beneficial to report the effect size (as illustrated in Figure 1) of the predictive model to further validate model stability. In addition, while cross-validation was performed, the specific method should be clearly stated. Was k-fold cross-validation used, or another approach?

***Answer:*** *We thank the reviewer for this important observation. We acknowledge that the sample size (n = 40) is limited for developing complex predictive models; therefore, the present study is framed as a proof-of-concept.*

*To further support model stability, we calculated the effect size (Hedges’ g) for the optimized SVR model, obtaining a value of −0.05 (95% CI: −0.36 to 0.26), indicating no meaningful systematic bias relative to indirect calorimetry.*

*Additionally, we have clarified in the Methods section that the validation approach was based on Leave-One-Out Cross-Validation (LOOCV), which is particularly suitable for small datasets, as it maximizes data utilization while providing an almost unbiased estimate of model performance.*

2. The model included body mass, stature, BMI, and BSA as predictors. Given that BMI and BSA are calculated from body mass and stature, correlations exist among these variables, potentially leading to multicollinearity. It is recommended that variance inflation factor (VIF) diagnostics be conducted to assess the extent of multicollinearity.

***Answer:*** *We thank the reviewer for this insightful comment. We agree that intrinsic correlations exist among body mass, stature, BMI, and body surface area, and VIF diagnostics confirmed elevated multicollinearity among these predictors.*

*However, the final model was based on Support Vector Regression (SVR) with a non-linear radial basis function (RBF) kernel, which is less sensitive to multicollinearity compared to traditional linear regression approaches. Specifically, SVR incorporates regularization (parameter C) and transforms the input space into a higher-dimensional feature space, allowing it to handle correlated predictors without compromising model stability or predictive performance.*

*To improve transparency, we have clarified this aspect in the Methods section and explicitly acknowledged the presence of multicollinearity and the rationale for using SVR as a robust alternative under these conditions.*

3. In the study, the dataset was randomly partitioned into an 80–20 split for model training and external validation. However, this procedure still constitutes internal hold-out validation. True external validation would require testing the model on an independent dataset from a separate group of athletes.

***Answer:*** *We agree with the reviewer; this point has been clarified in the discussion.*

4. The discussion section, the authors may consider discussing whether similar modeling approaches have been adopted in prior research and comparing their findings with existing studies. This would help clarify the theoretical contribution and practical implications of the present study.

***Answer:*** *We thank the reviewer for this suggestion. To the best of our knowledge, there are currently no studies applying a comparable machine-learning framework for RMR prediction specifically in professional soccer players. This has now been clarified in the Discussion section, where we also position our findings within the broader context of emerging data-driven approaches in sports science.*

**Responses to Reviewer #2:**

1. **Small Sample Size and Outdated Data (Critical Limitation)**

Problem: The study sample consists of only 40 individuals, with data collected in 2014 (referred to as the "Opening Tournament 2014"). This small sample size not only limits the training efficacy and generalizability of the machine learning model (even though the authors use learning curves to suggest no overfitting, the small sample remains a major constraint) but also raises questions about whether data nearly a decade old accurately reflects the physiological characteristics of current professional football players. Training methodologies, nutritional strategies, and athlete body composition have evolved significantly over the past decade.

Suggestions:

(1)In the discussion section, this must be addressed more profoundly and transparently as the primary limitation. Clearly state the impact of the small sample size and outdated data on the generalizability of the conclusions.

***Answer:*** *We thank the reviewer for this important observation. We have revised the Discussion section to explicitly acknowledge the small sample size and the use of data collected in 2014 as critical limitations. Their potential impact on generalizability and contemporary relevance is now discussed in greater depth.*

(2)Explicitly frame the machine learning model as a "pilot exploration," as already indicated in the title and text. Emphasize that its results require external validation in larger, multi-center, and more recent cohorts before any practical application can be considered.

***Answer:*** *We agree with the reviewer. The manuscript has been revised to clearly state that the machine-learning model should be considered exploratory. We now emphasize that external validation in independent and multicenter cohorts is required before any practical application.*

(3)If possible, attempt to contact the authors or the club to see if newer or additional data can be obtained to augment the sample. However, this is often difficult to achieve, so a frank acknowledgment of the limitations is the more realistic path forward.

***Answer:*** *We appreciate the reviewer’s suggestion. Unfortunately, it was not possible to obtain additional or more recent data from the club or the original data sources. As recommended, we have explicitly acknowledged this limitation in the revised manuscript and discussed its implications for the generalizability and contemporary relevance of the findings.*

2. **Lack of External Validation for the Machine Learning Model**

Problem:The model's superior performance is demonstrated solely based on an internal validation set (20% of the data) and cross-validation. In machine learning research, the absence of an independent external validation set is a critical weakness. This leaves the true generalizability of the model unknown, with a risk of overfitting to the specific characteristics of these 40 players. Although the authors examined learning curves, this does not substitute for external validation.

Suggestions:

(1) Explicit Differentiation:In the title, abstract, and conclusions, employ more cautious phrasing, such as "preliminary results suggest," "demonstrated strong performance in internal validation," etc., to avoid creating the impression that the model is ready for widespread application.

***Asnwer:*** *We thank the reviewer for this important suggestion. The title, abstract, and conclusions have been revised to adopt a more cautious and accurate tone. Specifically, we have introduced terms such as “preliminary” and clarified that the machine learning model demonstrated improved performance under internal validation conditions. We have also emphasized the exploratory nature of the findings and the need for external validation before practical application.*

(2) Future Research Direction:In the discussion section, emphasize "external validation" as the most critical next step for research. Concrete suggestions could be made that future studies should validate the model in professional player cohorts from different leagues, different countries, and different age groups.

***Answer:*** *We agree with the reviewer. The manuscript has been revised to clearly state that the machine-learning model should be considered exploratory. We now emphasize that external validation in independent and multicenter cohorts is required before any practical application.*

3. **Lack of Methodological Clarity**

Problem:The description of data preprocessing and feature engineering for the machine learning part is vague. For instance, how exactly were the "nonlinear and interaction terms" created? Which features were used? Similarly, the specific range and methods for hyperparameter tuning are not described, affecting the reproducibility of the study.

Suggestions:

(1) In the "Machine Learning Analysis" section, supplement the text with a table listing all features ultimately input into the model (e.g., weight, height, BMI, body surface area, and potentially constructed interaction terms like weight², weight × height).

***Answer:*** *We thank the reviewer for this important observation. We have substantially improved the transparency and reproducibility of the machine learning methodology. Specifically, we expanded the Data Preprocessing section to explicitly describe the feature engineering process, including the generation of nonlinear (e.g., squared terms) and interaction terms (e.g., body mass × stature).*

*In addition, we have incorporated a new table (Table X) in the Machine Learning Analysis section that details all input features used in the model, including primary (e.g., body mass, stretch stature, age), derived (BMI, BSA), nonlinear, and interaction variables, along with their formulas and rationale.*

*These additions provide a clear and reproducible description of the predictors included in the modeling process.*

(2) Provide a detailed description of the hyperparameter tuning process. For example, for the SVR model, specify the kernel function type (e.g., RBF kernel) and describe the range used for tuning parameters such as C, epsilon, and gamma using methods like GridSearch or RandomizedSearch, along with the final optimal values chosen. This would greatly enhance the scientific rigor of the study.

***Answer:*** *We appreciate the reviewer’s suggestion to improve methodological transparency. In response, we have added a dedicated subsection (Hyperparameter Optimization of the SVR Model) within the Machine Learning Analysis section.*

*In this subsection, we now explicitly describe the optimization procedure, including the use of GridSearchCV with 5-fold cross-validation, the selection of a radial basis function (RBF) kernel, and the explored parameter ranges for C, ε, and γ. Hyperparameter tuning was performed using 5-fold cross-validation, while final model validation was conducted using Leave-One-Out Cross-Validation (LOOCV) to maximize data utilization given the small sample size. We also report the final optimal values selected (C = 10, ε = 0.1, γ = ‘scale’).*

4. **Contradictory Presentation and Over-interpretation of Results**

Problem:The abstract mentions that the SVR model "reduced the error by 227 to 379 kcal/day." This value appears derived by subtracting the SVR's MAE from the "bias" of the traditional equations. This comparison is misleading. MAE measures the average absolute error at the individual level, while "bias" is the average difference (mean error). These are not the same concept, and directly subtracting them exaggerates the improvement. Furthermore, the SVR model's R² is only 0.169. Although this is better than the negative or near-zero values of the traditional equations, it still means the model can only explain approximately 17% of the variance in RMR, indicating its predictive capability is actually quite limited.

***Answer:*** *We agree with the reviewer. The conclusions have been revised to avoid overinterpretation of the machine learning model. We now explicitly state that the findings are preliminary and exploratory, and that the model should be considered a proof of concept rather than a ready-to-use tool. We have also highlighted the need for cautious interpretation and further validation in larger and independent cohorts.*

Suggestions:

(1) Rephrase the relevant descriptions in the abstract and conclusions. A more accurate statement would be: The SVR model's prediction error (MAE) was substantially lower than the average bias or RMSE of the traditional equations, demonstrating higher accuracy in individual prediction. Avoid directly subtracting values to derive a specific "reduction" range.
 ***Answer:*** *We thank the reviewer for identifying this important issue. The misleading comparison between MAE and bias has been removed from the abstract and conclusions. We have reformulated the results to provide a more appropriate interpretation, stating that the MAE of the SVR model was lower than the RMSE and average bias observed in traditional equations, without directly subtracting these metrics.*

(2) In the discussion, provide a balanced interpretation of the R² value of 0.169. Acknowledge its progress compared to the traditional equations, while candidly pointing out that a large amount of unexplained variance remains. This highlights the complexity of RMR and suggests that future work may need to incorporate more predictive factors (such as training load, hormonal levels, more precise body composition data, etc.).

***Answer:*** *We agree with the reviewer and have revised the manuscript accordingly. The abstract and conclusions now explicitly acknowledge that the SVR model explains a limited proportion of the variance (R² = 0.169). We have incorporated a more balanced interpretation, emphasizing both the relative improvement over traditional equations and the remaining unexplained variability.*

5. **Competing Interests Statement is Vague**

Problem:Some authors have affiliations with Breezing Co., the company that manufactures the device used in this study. Although the statement mentions "no financial compensation was received," "technical consulting and development support" in itself constitutes a potential conflict of interest that could influence the objectivity of the research. The statement claims this "did not influence the study outcomes," but this judgment should ultimately be made by the reviewers and readers.

Suggestion:

(1) It is recommended that the authors provide a clearer and more transparent competing interests statement. For example, they could specify the nature and duration of the consulting/support, and the measures taken to prevent bias (e.g., data analysis was performed by a third party not involved in the commercial collaboration). The current statement, "It did not influence the study outcomes," is overly subjective. PLOS has strict requirements regarding competing interests; vague language could potentially raise issues.

***Answer:*** *We thank the reviewer for this important comment. The Conflict of Interest statement has been revised to improve transparency and clarity. Specifically, we have provided a more precise description of the nature of the collaboration and removed subjective statements. Additionally, we have clarified that the statistical analyses were conducted by an author independent of this collaboration to minimize potential bias.*

**Minor Comments**

Writing and Grammar: The overall readability of the manuscript is acceptable, but there are minor grammatical errors and instances of unnatural phrasing. For example, the sentence in the abstract, "corresponding to a reduction of 227 to 379 kcal·day-1 based on the difference between its MAE and the observed biases," as previously noted, is inaccurately phrased and grammatically awkward. It is suggested that the authors ask a native English-speaking colleague or a professional editing service to polish the language of the full manuscript to enhance fluency and professionalism.

Clarity of Figures/Tables:

Table 3:The title is in English, but the header for the first column is "Modelo" (Spanish/Portuguese). This should be unified to English, i.e., "Model." Please review the entire manuscript to ensure all elements are in English.

***Answer:*** *Corrected. The term has been revised to “Model”, and the manuscript has been carefully checked to ensure consistency in the use of English throughout.*

Figure 1:The figure legend mentions "RMSE and bias values," but these numerical values are not shown in the figure itself; only effect sizes are displayed. It is recommended to either annotate the RMSE and bias for key equations directly on the figure or provide this information in a separate table for more complete presentation.

***Answer:*** *We thank the reviewer for this valuable suggestion. To improve the clarity and completeness of the results, we have added a new table (Table X) presenting the RMSE and bias values alongside the effect sizes (Hedges’ g) for all predictive equations and the optimized SVR model.*

*This table complements Figure 1 and allows for a more comprehensive interpretation of both the magnitude of differences (effect size) and the predictive accuracy (RMSE and bias). The corresponding reference to this table has been included in the Results section.*

Reference Format: Please check the reference format to ensure it fully complies with the journal requirements of PLOS One. For instance, in reference number 5, the journal name is written in all capitals; this should be corrected to the standard format.

*Answer: We thank the reviewer for this comment. The reference list has been carefully revised to ensure full compliance with PLOS ONE formatting guidelines. Although we did not identify the specific issue mentioned, all references have been standardized and minor inconsistencies have been corrected.*

Data Availability Statement: The authors state, "All relevant data are within the manuscript and its Supporting Information files." It is recommended that the authors upload the anonymized data for the 40 players used to train the SVR model (age, height, weight, IC-measured RMR values) as a Supporting Information file. This would genuinely make the data open, which is a core policy requirement of PLOS journals.

***Answer:*** *We thank the reviewer for this suggestion. The anonymized dataset used in this study (including age, height, body mass, and RMR values) has been deposited in a public repository (Figshare). The DOI (*[*https://doi.org/10.6084/m9.figshare.31872430*](https://doi.org/10.6084/m9.figshare.31872430)*) has been included in the revised Data Availability Statement, ensuring full transparency and enabling replication of the study findings.*

**Additional Comments**

Introduction Section The introduction effectively establishes the background and necessity of the study. It could be slightly streamlined by reducing general knowledge about energy expenditure in football and focusing more on the limitations of existing RMR prediction equations in athletic populations. This would better highlight the innovative aspects and urgency of this research.

***Answer:*** *We thank the reviewer for this valuable suggestion. The Introduction has been revised to improve focus and clarity by emphasizing the limitations of traditional RMR predictive equations in athletic populations, while reducing the relative weight of general background information on soccer. These changes aim to better highlight the rationale, innovation, and relevance of the study.*

Discussion Section: The discussion provides a thorough analysis of why the traditional equations fail. However, when discussing the advantages of the ML model, greater caution is needed to avoid giving the erroneous impression that the "ML model is good enough." Its "preliminary" nature and the need for "validation" must be repeatedly emphasized. Concurrently, future research directions could be made more concrete. For example, proposing an ideal study design: a prospective, multi-center cohort study collecting multi-dimensional data including DXA body composition, training load, and hormonal levels to build and validate more robust prediction models.

***Answer:*** *We thank the reviewer for this suggestion. The Discussion section has been revised to adopt a more cautious interpretation of the machine-learning model. We now explicitly emphasize its preliminary nature, the need for external validation, and provide clearer and more concrete directions for future research, including the use of larger, multicenter cohorts and the incorporation of additional physiological variables.*
