# Supplementary material for: Estimation of resting metabolic rate in professional soccer players: A cross-sectional study comparing traditional predictive equations and a preliminary machine learning model against indirect calorimetry
Source: PLoS One. 2026 Aug 12;21(8):e0354973. doi: 10.1371/journal.pone.0354973 (PMC13465844; doi:10.1371/journal.pone.0354973)
Supplement: Supplementary file 2 [file pone.0354973.s004.docx]

**Response to Reviewers**

*Manuscript: “Estimation of Resting Metabolic Rate in Professional Soccer Players: A Cross-Sectional Study Comparing Traditional Predictive Equations and a Preliminary Machine Learning Model Against Indirect Calorimetry”*

**Dear Editor and Reviewers,**

We sincerely thank the editor and the reviewers for the careful and constructive evaluation of our manuscript. The comments have helped us improve the rigor, transparency, and internal consistency of the work. We have addressed every point raised, and we respond to each of them in detail below. For clarity, the reviewers’ comments are reproduced in italics, followed by our response. All corresponding modifications in the revised manuscript are highlighted in yellow.

**Comment 1.**

*The description of the machine-learning validation is unclear and internally inconsistent: the response letter referred to leave-one-out cross-validation (LOOCV), whereas the manuscript describes an 80/20 split with 5-fold cross-validation. The respective roles of these procedures should be distinguished, the risk of optimistic performance should be acknowledged (or a nested validation used), and it should be stated whether preprocessing, outlier removal, scaling, and feature engineering were performed within the training folds (to rule out data leakage).*

**Response.** We have clarified the validation strategy and rewritten the relevant parts of the Methods (subsections “Data Preprocessing” and “Study Design and Population”). The revised text now states explicitly that (i) model development followed a single random partition of the dataset into a training subset (80%) and a held-out internal-validation subset (20%); (ii) hyperparameters were tuned by 5-fold cross-validation applied only within the training subset; and (iii) the held-out subset was used once to estimate the performance metrics reported in the manuscript. We have also removed any reference to leave-one-out cross-validation, which was not used to obtain the final estimates, thereby resolving the inconsistency with the previous response letter.

To rule out data leakage, the preprocessing description has been rewritten to make the order of operations explicit: the dataset is partitioned first, and all subsequent preprocessing steps (outlier screening and RobustScaler standardization) are fitted exclusively on the training data and then applied, unchanged, to the held-out subset. Finally, because both hyperparameter tuning and performance estimation are derived from a single small dataset (n = 40) and no external validation set was available, we now state that the resulting metrics may be optimistically biased and should be interpreted as exploratory (proof-of-concept) rather than as confirmed estimates of out-of-sample accuracy.

**Comment 2.**

*The conclusions regarding the SVR model are too strong given the small sample, the absence of external validation, and the low coefficient of determination (R² = 0.169). Statements such as “adequate stability and generalization,” “practical and reliable alternative,” “most reliable computational approach,” and “systematically increases the accuracy” should be softened, and the model should be reframed as exploratory or proof-of-concept.*

**Response.** We fully agree and have softened every statement that implied confirmed reliability or generalizability. Across the Results, the description of Figure 3, and the Discussion, the relevant expressions have been reframed. The model is now described as having shown the most favorable internal-validation results among the algorithms evaluated, while we explicitly note the low coefficient of determination (R² = 0.169), which indicates that most of the between-individual variability in RMR remained unexplained, as well as the small sample and the absence of external validation.

In particular, “adequate stability and generalization” has been replaced by an explicit statement that the small sample and the lack of external validation preclude any firm conclusion regarding stability or generalization; “practical and reliable alternative” has been replaced by a statement that external validation in larger, independent cohorts is required before any practical application can be considered; and the claim that the approach “systematically increases the accuracy” has been reframed as an exploratory observation limited to the present internal-validation analysis. Throughout, the model is now consistently described as exploratory (proof-of-concept).

**Comment 3.**

*There are numerical and interpretive inconsistencies between the text and the tables. The Results state that FAO/WHO, Henry, and Valencia had the lowest relative errors, which contradicts Table 2; the reported bias range omits the smallest value; the term “best-performing” is used inconsistently; and the Pearson correlation values differ between paragraphs. All numerical statements should be checked against Tables 2–5.*

**Response.** We thank the reviewer for identifying these inconsistencies and apologize for the errors. We have re-checked every numerical statement against Tables 2–5 and corrected them as follows:

• Table 2 (relative errors): the ranking has been corrected. The equation of Owen et al. (1987) showed the lowest relative error, followed by those of Kim et al. (2015), Mifflin et al. (1990), and Müller et al. (2004), whereas FAO/WHO (1985), Harris and Benedict (1918), and Wong et al. (2012) showed the largest discrepancies. The previous text, which listed FAO/WHO, Henry, and Valencia as having the lowest errors, was incorrect (FAO/WHO in fact showed the highest relative error, 36.4%).

• Table 3 (bias): the bias range has been corrected from “154 to 452” to “48 to 452 kcal·day⁻¹” so as to include the smallest bias, that of Owen et al. (47.7 kcal·day⁻¹).

• Definition of “best-performing”: we now define this consistently. the highest intraclass and concordance coefficients corresponded to Henry's equation (2005) [22] (ICC = CCC = 0.030), whereas the lowest bias and RMSE corresponded to Owen's equation (1987) [29] (bias = 47.7 kcal·day⁻¹; RMSE = 355.8 kcal·day⁻¹); however, agreement remained poor for all equations. The same wording is now used in the Results and in the Discussion.

• Pearson correlation: the contradictory values in the second concordance paragraph have been harmonized to r = −0.205 to 0.080 (ρ > 0.05), consistent with the first paragraph and with Table 3.

• Conclusions: the overestimation range and the average bias have been corrected to 48 to 452 kcal·day⁻¹ and approximately 290 kcal·day⁻¹, respectively (the previous average of 315 kcal·day⁻¹ had inadvertently excluded the lowest-bias equation).

**Comment 4.**

*Several predictors (BMI, BSA, squared body mass, and the body mass × stature interaction) are derived from body mass and stature and are therefore redundant, which is problematic for n = 40. Variance inflation factor (VIF) diagnostics should be reported, or a sensitivity analysis using only primary variables performed, or it should be stated explicitly that no inference is made about predictor importance.*

**Response.** We agree. Because the derived predictors (body mass index, body surface area, squared body mass, and the body mass × stature interaction) are mathematical functions of body mass and stature, the predictor set is collinear by construction. We now report that the variance inflation factors confirmed severe multicollinearity, with values for the derived terms well above the conventional threshold of 10. Accordingly, we have added an explicit statement (in the feature-engineering description) that, given this redundancy and the small sample (n = 40), the models were used solely for prediction and that no inference is made regarding the relative importance or the independent contribution of individual predictors.

**Comment 5.**

*It should be reported how many observations were removed as outliers by the IQR criterion; with n = 40 this is material. It should also be clarified whether the metrics remain robust when all observations are retained.*

**Response.** We have clarified the outlier procedure in the “Data Preprocessing” subsection. Application of the IQR criterion did not flag any observation as an outlier; consequently, all 40 participants were retained, and the metrics reported in the manuscript correspond to the complete sample. The question of robustness when all observations are retained is therefore addressed directly, since no observations were excluded.

**Comment 6.**

*The competing-interests statement should be made more transparent: the subjective assertion that the relationship did not influence the study outcomes should be removed, and the nature of the Breezing consulting relationship, the authors involved, the safeguards adopted, and the identity of the person who performed the statistical analysis should be specified.*

**Response.** We have expanded the Conflict of Interest statement to improve transparency. The subjective assertion regarding outcomes not being influenced has been removed. The statement now specifies that the consulting and development support provided by C.A.H.-A. and C.O.R.-G. concerned the indirect-calorimetry methodology underlying the Breezing® device, and that the company had no role in the study design, in the collection, analysis, or interpretation of the data, or in the decision to submit the manuscript for publication; the device was used solely as the reference instrument for indirect calorimetry. As a safeguard against potential bias, the statistical analyses were conducted by an independent author (R.Y.-S.) who is not part of this collaboration.

**Comment 7.**

*The data were collected in 2014, whereas the ethics approval code (CEI-062020-01) corresponds to 2020. It should be clarified whether the study was prospective or retrospective, and confirmed that the informed consent covered the use of the data for research and their public sharing, particularly for participants who were minors.*

**Response.** We have clarified the temporal and consent aspects in the Ethics statement. The data were originally collected in 2014 within the framework of routine sports-nutrition assessment; ethical approval for the retrospective analysis and for the publication of these anonymized data was subsequently granted by the committee (Code: CEI-062020-01), which explains the apparent discrepancy in dates. We have also clarified that the informed consent signed by the participants, and the parental consent obtained for those aged < 18 years, explicitly covered the use of the collected data for research purposes and their subsequent anonymized public sharing.

**Comment 8.**

*Please submit a clean version of the manuscript and remove the earlier claim regarding a reduction of 227 to 379 kcal/day.*

**Response.** A clean version has been prepared. The earlier claim regarding a reduction of 227 to 379 kcal/day has been removed, and the manuscript no longer contains conflicting statements on this point. In the present file, only the changes introduced in this revision are highlighted in yellow, so that the revised content can be readily identified.

We believe that these revisions have substantially improved the clarity, rigor, and transparency of the manuscript, and we are grateful to the reviewers for their valuable and constructive input. We remain at your disposal for any further clarification.

Sincerely,

José Francisco López-Gil

*Corresponding author, on behalf of all co-authors*
