# Supplementary material for: Estimation of resting metabolic rate in professional soccer players: A cross-sectional study comparing traditional predictive equations and a preliminary machine learning model against indirect calorimetry
Source: PLoS One. 2026 Aug 12;21(8):e0354973. doi: 10.1371/journal.pone.0354973 (PMC13465844; doi:10.1371/journal.pone.0354973)
Supplement: Supplementary file 3 [file pone.0354973.s005.docx]

**Response to Reviewers**

**Manuscript ID: PON-D-26-02771R2**

Title: Estimation of basal metabolic rate in professional soccer players: a cross-sectional study comparing traditional predictive equations and a preliminary machine-learning model with indirect calorimetry.

Dear Dr. Jaafar and Reviewer #2,

We thank the academic editor and reviewer #2 for their thorough and constructive evaluation. We have revised the manuscript to address all the points raised. All changes made in this revision are highlighted in yellow in the revised manuscript: “Manuscript_R4_corrected_highlighted.” We respond to each comment below (the reviewer’s text appears in italics).

**Reviewer #2 - Major Comments**

***Reviewer comment.*** *Possible inconsistency in the submitted file: the reviewer PDF appeared to contain a response section referring to a different manuscript on football analytics, PCA/SHAP, and offensive/defensive player profiling.*

**Response.** We apologize for this compilation error. We have verified that both the response letter and the manuscript now correspond exclusively to the present study on resting metabolic rate in soccer players; no material relating to any other manuscript is included. We have re-uploaded a single, coherent set of files (Manuscript, Revised manuscript with tracked changes, and this Response to Reviewers).

***Reviewer comment.*** *The machine-learning model should remain clearly framed as exploratory; with n = 40 and R² = 0.169 the model must not be presented as validated, generalizable, or ready for applied use.*

**Response.** We agree and have kept the SVR model described consistently as a preliminary, internal-validation, proof-of-concept analysis throughout the Abstract, Methods, Results, the Figure 3 legend, the Discussion and the Conclusions. We explicitly state that the held-out performance "may be optimistically biased", that the low *R*² (0.169) leaves most between-individual variability unexplained, and that external validation in larger, independent cohorts is required before any practical use. Earlier wording implying stability, generalization, reliability, or readiness for practice has been removed.

***Reviewer comment.*** *The validation strategy and prevention of data leakage must be fully transparent (split ratio; whether tuning, preprocessing, outlier detection, scaling and feature engineering were fitted only on training data; whether the held-out set was used only once; acknowledgement of optimistic bias).*

**Response.** The Methods now state explicitly: a single 80/20 train/validation split; hyperparameter tuning by 5-fold cross-validation performed only within the training subset; preprocessing, outlier screening and RobustScaler scaling fitted exclusively on the training data and then applied unchanged to the held-out set; the held-out set used only once for the final internal-validation estimate; and an explicit acknowledgement that, given the small sample, these estimates may be optimistically biased. We also note that the engineered terms (squared body mass and the body mass × stature interaction) are deterministic transformations and therefore cannot leak information.

***Reviewer comment.*** *Multicollinearity among predictors must be handled cautiously: report VIF diagnostics or state clearly that the model is for prediction only and no inference is made about individual predictors.*

**Response.** We have done both. The Methods now report the variance inflation factors computed from the data, approximately 3.5 × 10⁵ (body mass), 6.6 × 10⁵ (body surface area), 1.8 × 10⁵ (body mass × stature), 1.1 × 10⁵ (squared body mass) and 1.6 × 10³ (body mass index), versus 1.7 for age, confirming that the predictor set is collinear by construction. We state explicitly that the models are used solely for prediction and that no inference is made regarding the relative importance or independent contribution of any predictor.

***Reviewer comment.*** *Indirect calorimetry procedures require sufficient methodological detail (calibration, fasting, prior-exercise restriction, caffeine/stimulant control, environmental conditions, rest period, measurement duration, steady-state criteria, handling of implausible respiratory quotient values).*

**Response.** We thank the reviewer for this important comment regarding methodological detail in indirect calorimetry procedures. Several of the requested elements were already reported in the manuscript under the “Data sources and measurements” section, including an overnight fast of 7–8 h, a minimum of 12 h abstention from exercise, and a 48 h restriction from alcohol, stimulants, food, and dietary supplements (including coffee, tea, chocolate, carbonated beverages, and energy drinks) prior to testing.

In the Resting metabolic rate subsection, we have now ensured that all remaining methodological requirements are explicitly reported in a single location to improve clarity and completeness. Specifically, we confirm device calibration prior to each test, a 5–10 min familiarization period, testing in the supine position, calculation using the Weir equation, a total measurement window of approximately 12–15 min, environmental control (quiet, thermoneutral room), application of steady-state criteria based on the Academy of Nutrition and Dietetics best-practice guidelines, and the screening of respiratory exchange ratio (RER) values within the physiologically plausible range (0.70–1.00).

***Reviewer comment.*** *Numerical consistency across text, tables and figures must be ensured, and "best-performing" must be defined consistently.*

**Response.** We have reconciled all numerical statements. Specifically: (i) the limits of agreement for the Hannon equation in Table 3 were corrected (the signs had been inverted; they now read −321.2 to 1073.4, consistent with a positive mean bias of +376.1); (ii) the concordance-coefficient range in the text was corrected to −0.091 to 0.030 to match Table 3 (Lin's CCC for Kim = −0.091); (iii) the statement that the Owen equation showed "the widest limits of agreement" was corrected, since its span (~1,400 kcal·day⁻¹) is comparable to, not wider than, the other equations; the text now reflects that Owen combines the lowest bias with wide, imprecise limits; (iv) the FAO/WHO relative error in Table 2 was harmonized to 36.4% to match the text and Abstract; (v) bias and error values are now reported to one decimal place consistently across the text, Table 3 and Table 4 (e.g., Owen bias 47.8 kcal·day⁻¹); and (vi) the Figure 1 narrative was aligned with the Hedges' g ranking in Table 4. "Best agreement" is defined explicitly by the two relevant criteria, distinguishing the equation with the highest ICC/CCC (Henry) from that with the lowest bias and RMSE (Owen), while noting that agreement remained poor for all equations.

***Reviewer comment.*** *The ethics and consent timeline must be clarified (2014 data collection versus a later ethics-approval code), and informed/parental consent for public sharing of anonymized data must be confirmed.*

**Response.** The Ethics statement clarifies that data were collected during the 2014 Opening Tournament and that the institutional committee (Code CEI-062020-01) granted approval for the retrospective analysis and for the publication of anonymized data. The statement confirms that all participants provided written informed consent, including parental consent for participants under 18 years of age, covering both the research use and the public sharing of anonymized data.

**Minor Comments**

***Reviewer comment.*** *The conclusion that traditional equations are "not adequate" may be too categorical; "showed poor agreement in this sample" would be preferable.*

**Response.** We have softened the Abstract conclusion to "Traditional equations showed poor agreement with indirect calorimetry in this sample of soccer players", and the Discussion/Conclusions consistently frame the limitation as poor agreement and limited external validity rather than categorical inadequacy.

***Reviewer comment.*** *Units should be standardized (kcal/day vs kcal·day⁻¹).*

**Response.** The manuscript, figures & tables now uses kcal·day⁻¹ throughout, including the Table 5 column headers (MAE and RMSE) and the corresponding sentence, which previously used bare "kcal". We also standardized the oxygen-consumption notation to VO₂.

***Reviewer comment.*** *The Abstract should retain the cautious description of the SVR model and avoid implying external validity.*

**Response.** The Abstract describes the model as a preliminary machine-learning approach and proof of concept, reports the low *R*² (0.169), and states that predictive capacity remains limited and that external validation is required. No claim of external validity is made.

***Reviewer comment.*** *The competing-interests statement should identify any relationship with the indirect-calorimetry device/company, the authors involved, the company role, and who performed the statistical analyses.*

**Response.** The Competing Interests statement identifies the relationship with Breezing Co., names the authors involved (C.A.H.-A. and C.O.R.-G.), describes the nature of the relationship (technical consulting on the indirect-calorimetry methodology) and the honoraria received, states that the company had no role in study design, data collection, analysis, interpretation or the decision to publish, and confirms that the statistical analyses were performed independently by R.Y.-S.

***Reviewer comment.*** *The Data Availability Statement should provide a clear, accessible link and specify whether the shared dataset is raw, processed, or anonymized.*

**Response.** The Data Availability Statement provides the Figshare DOI link and now specifies that the deposited dataset is the anonymized, processed, individual-level dataset containing all data needed to reproduce the results.

We believe these revisions address all the points raised and we remain at your disposal for any further clarification.

Sincerely,

The Authors
