# Supplementary material for: Estimation of resting metabolic rate in professional soccer players: A cross-sectional study comparing traditional predictive equations and a preliminary machine learning model against indirect calorimetry
Source: PLoS One. 2026 Aug 12;21(8):e0354973. doi: 10.1371/journal.pone.0354973 (PMC13465844; doi:10.1371/journal.pone.0354973)
Supplement: Supplementary file 4 [file pone.0354973.s002.doc]

I appreciate the authors’ substantial revisions. The manuscript is now more cautious in framing the machine-learning component, the limitations are more explicit, methodological details have been expanded, and the dataset has been made available through Figshare. Overall, the revision has improved the manuscript considerably.

However, several issues should still be addressed before acceptance.

1. The machine-learning validation procedure remains unclear and partly inconsistent. The response letter states that final validation was based on LOOCV, whereas the manuscript still describes an 80/20 training/internal validation split and 5-fold cross-validation for hyperparameter tuning. Please clearly distinguish the roles of the 80/20 split, 5-fold CV, and LOOCV. If hyperparameter tuning and final validation were performed on the same small dataset, the authors should acknowledge the risk of optimistic performance estimation or, preferably, use a nested validation framework. The manuscript should also state whether preprocessing steps, outlier removal, scaling, and feature engineering were performed within each training fold to avoid data leakage.

2. Some claims about the optimized SVR model remain too strong given the small sample size, lack of external validation, and low R² value (0.169). Phrases such as “adequate stability and generalization,” “practical and reliable alternative,” “most reliable computational approach,” and “systematically increases the accuracy” should be softened. A more appropriate framing would be that the SVR model showed better internal-validation performance and should be regarded as exploratory or proof-of-concept.

3. There are remaining numerical and interpretive inconsistencies. For example, the Results section states that FAO/WHO, Henry, and Valencia had the lowest relative errors, but Table 2 appears to show Owen as the lowest relative error, followed by Kim, Mifflin, and Müller. The manuscript also states that biases ranged from 154 to 452 kcal/day, which appears to omit Owen’s lower bias. Please check all statements against Tables 2–5 and ensure that “best-performing” is defined consistently, e.g., by bias, RMSE, ICC/CCC, or relative error.

4. The manuscript should report the VIF diagnostics that were mentioned in the response letter. Because BMI, BSA, body mass squared, and body mass × stature are mathematically derived from body mass and stature, the predictor set is highly redundant for a sample of only 40 athletes. The authors should either provide a sensitivity analysis using only primary anthropometric variables or explicitly state that no inference about predictor importance is being made.

5. Please report how many observations, if any, were removed as outliers using the IQR criterion. With n = 40, outlier removal can materially affect model performance. The authors should also clarify whether the reported metrics are robust when all observations are retained.

6. The competing interests statement should be made fully transparent. The submission materials still include language indicating that the collaboration “did not influence the study outcomes,” which is subjective. Please specify the nature of the technical consulting/development support for Breezing Co., the authors involved, and the safeguards used to reduce bias, including who conducted the statistical analysis.

7. The ethics statement needs clarification. Data were collected in 2014, whereas the ethics approval code appears to refer to 2020. Please clarify whether approval was prospective or retrospective and confirm that participant consent covered research use and public data sharing, especially if any participants were minors.

8. Finally, the submission package appears to contain multiple manuscript versions, including a clean revised manuscript and a tracked-changes version that still includes some older wording. Please ensure that the final manuscript file is the revised clean version only and that outdated wording, especially the previous “reduction of 227 to 379 kcal/day” claim, is removed from the final article.

In summary, the revised manuscript is much improved and the main contribution is potentially useful. I would support publication after these remaining methodological clarifications, consistency checks, and tone adjustments are completed.
