# Supplementary material for: Estimation of resting metabolic rate in professional soccer players: A cross-sectional study comparing traditional predictive equations and a preliminary machine learning model against indirect calorimetry
Source: PLoS One. 2026 Aug 12;21(8):e0354973. doi: 10.1371/journal.pone.0354973 (PMC13465844; doi:10.1371/journal.pone.0354973)
Supplement: Peer review history

# Peer review: Decision Letter 0

**DOI:** 10.1371/journal.pone.0354973.r001

**Authors:** Zulkarnain Jaafar

17 Mar 2026

PONE-D-26-02771

Estimation of Resting Metabolic Rate in Professional Soccer Players: A Cross-Sectional Study Comparing Traditional Predictive Equations and a Pilot Machine Learning Model Against Indirect Calorimetry

PLOS One

Dear Dr. López-Gil,

Thank you for submitting your manuscript to PLOS ONE. After careful consideration, we feel that it has merit but does not fully meet PLOS ONE’s publication criteria as it currently stands. Therefore, we invite you to submit a revised version of the manuscript that addresses the points raised during the review process.

==============================

**ACADEMIC EDITOR: Dear Author, please revise your manuscript based on the comments provided by the reviewers.**

==============================

Please submit your revised manuscript by May 01 2026 11:59PM. If you will need more time than this to complete your revisions, please reply to this message or contact the journal office at plosone@plos.org. When you're ready to submit your revision, log on to https://www.editorialmanager.com/pone/ and select the 'Submissions Needing Revision' folder to locate your manuscript file.

Please include the following items when submitting your revised manuscript:

- A letter that responds to each point raised by the academic editor and reviewer(s). You should upload this letter as a separate file labeled 'Response to Reviewers'.
- A marked-up copy of your manuscript that highlights changes made to the original version. You should upload this as a separate file labeled 'Revised Manuscript with Track Changes'.
- An unmarked version of your revised paper without tracked changes. You should upload this as a separate file labeled 'Manuscript'.

If you would like to make changes to your financial disclosure, please include your updated statement in your cover letter. Guidelines for resubmitting your figure files are available below the reviewer comments at the end of this letter.

If applicable, we recommend that you deposit your laboratory protocols in protocols.io to enhance the reproducibility of your results. Protocols.io assigns your protocol its own identifier (DOI) so that it can be cited independently in the future. For instructions see: https://journals.plos.org/plosone/s/submission-guidelines#loc-laboratory-protocols. Additionally, PLOS ONE offers an option for publishing peer-reviewed Lab Protocol articles, which describe protocols hosted on protocols.io. Read more information on sharing protocols at https://plos.org/protocols?utm_medium=editorial-email&utm_source=authorletters&utm_campaign=protocols.

We look forward to receiving your revised manuscript.

Kind regards,

Zulkarnain Jaafar

Academic Editor

PLOS One

Journal Requirements:

When submitting your revision, we need you to address these additional requirements.

1. Please ensure that your manuscript meets PLOS ONE's style requirements, including those for file naming. The PLOS ONE style templates can be found at

https://journals.plos.org/plosone/s/file?id=wjVg/PLOSOne_formatting_sample_main_body.pdf and

https://journals.plos.org/plosone/s/file?id=ba62/PLOSOne_formatting_sample_title_authors_affiliations.pdf

2. Please note that PLOS One has specific guidelines on code sharing for submissions in which author-generated code underpins the findings in the manuscript. In these cases, all author-generated code must be made available without restrictions upon publication of the work. Please review our guidelines at https://journals.plos.org/plosone/s/materials-and-software-sharing#loc-sharing-code and ensure that your code is shared in a way that follows best practice and facilitates reproducibility and reuse.

3. Please include a complete copy of PLOS’ questionnaire on inclusivity in global research in your revised manuscript. Our policy for research in this area aims to improve transparency in the reporting of research performed outside of researchers’ own country or community. The policy applies to researchers who have travelled to a different country to conduct research, research with Indigenous populations or their lands, and research on cultural artefacts. The questionnaire can also be requested at the journal’s discretion for any other submissions, even if these conditions are not met.  Please find more information on the policy and a link to download a blank copy of the questionnaire here: https://journals.plos.org/plosone/s/best-practices-in-research-reporting. Please upload a completed version of your questionnaire as Supporting Information when you resubmit your manuscript.

4. We note that your Data Availability Statement is currently as follows: “All relevant data are within the manuscript and its Supporting Information files.”

Please confirm at this time whether or not your submission contains all raw data required to replicate the results of your study. Authors must share the “minimal data set” for their submission. PLOS defines the minimal data set to consist of the data required to replicate all study findings reported in the article, as well as related metadata and methods (https://journals.plos.org/plosone/s/data-availability#loc-minimal-data-set-definition).

For example, authors should submit the following data:

- The values behind the means, standard deviations and other measures reported;

- The values used to build graphs;

- The points extracted from images for analysis.

Authors do not need to submit their entire data set if only a portion of the data was used in the reported study.

If your submission does not contain these data, please either upload them as Supporting Information files or deposit them to a stable, public repository and provide us with the relevant URLs, DOIs, or accession numbers. For a list of recommended repositories, please see https://journals.plos.org/plosone/s/recommended-repositories.

If there are ethical or legal restrictions on sharing a de-identified data set, please explain them in detail (e.g., data contain potentially sensitive information, data are owned by a third-party organization, etc.) and who has imposed them (e.g., an ethics committee). Please also provide contact information for a data access committee, ethics committee, or other institutional body to which data requests may be sent. If data are owned by a third party, please indicate how others may request data access.

5. PLOS requires an ORCID iD for the corresponding author in Editorial Manager on papers submitted after December 6th, 2016. Please ensure that you have an ORCID iD and that it is validated in Editorial Manager. To do this, go to ‘Update my Information’ (in the upper left-hand corner of the main menu), and click on the Fetch/Validate link next to the ORCID field. This will take you to the ORCID site and allow you to create a new iD or authenticate a pre-existing iD in Editorial Manager.

6. If the reviewer comments include a recommendation to cite specific previously published works, please review and evaluate these publications to determine whether they are relevant and should be cited. There is no requirement to cite these works unless the editor has indicated otherwise.

[Note: HTML markup is below. Please do not edit.]

Reviewers' comments:

Reviewer's Responses to Questions

**Comments to the Author**

1. Is the manuscript technically sound, and do the data support the conclusions?

The manuscript must describe a technically sound piece of scientific research with data that supports the conclusions. Experiments must have been conducted rigorously, with appropriate controls, replication, and sample sizes. The conclusions must be drawn appropriately based on the data presented.

Reviewer #1: Partly

Reviewer #2: No

**********

2. Has the statistical analysis been performed appropriately and rigorously?

Reviewer #1: Yes

Reviewer #2: No

**********

3. Have the authors made all data underlying the findings in their manuscript fully available?

The PLOS Data policy requires authors to make all data underlying the findings described in their manuscript fully available without restriction, with rare exception (please refer to the Data Availability Statement in the manuscript PDF file). The data should be provided as part of the manuscript or its supporting information, or deposited to a public repository. For example, in addition to summary statistics, the data points behind means, medians and variance measures should be available. If there are restrictions on publicly sharing data—e.g. participant privacy or use of data from a third party—those must be specified.

Reviewer #1: Yes

Reviewer #2: No

**********

4. Is the manuscript presented in an intelligible fashion and written in standard English?

PLOS ONE does not copyedit accepted manuscripts, so the language in submitted articles must be clear, correct, and unambiguous. Any typographical or grammatical errors should be corrected at revision, so please note any specific errors here.

Reviewer #1: Yes

Reviewer #2: No

**********

5. Review Comments to the Author

Please use the space provided to explain your answers to the questions above. You may also include additional comments for the author, including concerns about dual publication, research ethics, or publication ethics. (Please upload your review as an attachment if it exceeds 20,000 characters)

Reviewer #1: This study evaluated the agreement between twelve traditional RMR predictive equations and indirect calorimetry (IC) measurements in professional male soccer players, and introduced a machine learning approach (Support Vector Regression) to test prediction accuracy of athletes’ RMR. My comments and suggestions are presented as follows.

1)The machine learning sample consisted of 40 cases, which may be insufficient for constructing a robust predictive model (as acknowledged by the authors in the limitations section). A small sample size increases the risk of overfitting. Although the study conducted cross-validation and learning curve analyses, if feasible, it would be beneficial to report the effect size (as illustrated in Figure 1) of the predictive model to further validate model stability. In addition, while cross-validation was performed, the specific method should be clearly stated. Was k-fold cross-validation used, or another approach?

2)The model included body mass, stature, BMI, and BSA as predictors. Given that BMI and BSA are calculated from body mass and stature, correlations exist among these variables, potentially leading to multicollinearity. It is recommended that variance inflation factor (VIF) diagnostics be conducted to assess the extent of multicollinearity.

3)In the study, the dataset was randomly partitioned into an 80–20 split for model training and external validation. However, this procedure still constitutes internal hold-out validation. True external validation would require testing the model on an independent dataset from a separate group of athletes.

4)The discussion section, the authors may consider discussing whether similar modeling approaches have been adopted in prior research and comparing their findings with existing studies. This would help clarify the theoretical contribution and practical implications of the present study.

Reviewer #2: Manuscript Number: PONE-D-26-02771

Title: Estimation of Resting Metabolic Rate in Professional Soccer Players: A Cross-Sectional Study Comparing Traditional Predictive Equations and a Pilot Machine Learning Model Against Indirect Calorimetry

Abstract

This study evaluated the accuracy of 12 traditional resting metabolic rate (RMR) prediction equations in professional football (soccer) players by comparing them with indirect calorimetry (IC). The findings revealed that all traditional equations performed poorly, systematically overestimating RMR. In contrast, the research team developed an optimized Support Vector Regression (SVR) machine learning model, which demonstrated significantly superior predictive accuracy compared to the traditional equations (MAE: 169.3 kcal/day). These results set the stage for a discussion of the study's overall impact and limitations.

Overall Impression

The chosen topic holds significant practical importance, as it directly addresses a core challenge in sports nutrition: accurately assessing the energy requirements of athletes. Introducing machine learning methods to this field represents noteworthy methodological innovation. While the study offers valuable insights, it faces some limitations, including sample size, data age, methodological details, and result interpretation, which may affect the generalizability and immediate practical applicability of the model.

Review Conclusion

Major Revision

Major Comments

Below are the key issues that require the authors' focused revision and response:

1. Small Sample Size and Outdated Data (Critical Limitation)

Problem: The study sample consists of only 40 individuals, with data collected in 2014 (referred to as the "Opening Tournament 2014"). This small sample size not only limits the training efficacy and generalizability of the machine learning model (even though the authors use learning curves to suggest no overfitting, the small sample remains a major constraint) but also raises questions about whether data nearly a decade old accurately reflects the physiological characteristics of current professional football players. Training methodologies, nutritional strategies, and athlete body composition have evolved significantly over the past decade.

Suggestions:

(1)In the discussion section, this must be addressed more profoundly and transparently as the primary limitation. Clearly state the impact of the small sample size and outdated data on the generalizability of the conclusions.

(2)Explicitly frame the machine learning model as a "pilot exploration," as already indicated in the title and text. Emphasize that its results require external validation in larger, multi-center, and more recent cohorts before any practical application can be considered.

(3)If possible, attempt to contact the authors or the club to see if newer or additional data can be obtained to augment the sample. However, this is often difficult to achieve, so a frank acknowledgment of the limitations is the more realistic path forward.

2. Lack of External Validation for the Machine Learning Model

Problem:The model's superior performance is demonstrated solely based on an internal validation set (20% of the data) and cross-validation. In machine learning research, the absence of an independent external validation set is a critical weakness. This leaves the true generalizability of the model unknown, with a risk of overfitting to the specific characteristics of these 40 players. Although the authors examined learning curves, this does not substitute for external validation.

Suggestions:

(1) Explicit Differentiation:In the title, abstract, and conclusions, employ more cautious phrasing, such as "preliminary results suggest," "demonstrated strong performance in internal validation," etc., to avoid creating the impression that the model is ready for widespread application.

(2) Future Research Direction:In the discussion section, emphasize "external validation" as the most critical next step for research. Concrete suggestions could be made that future studies should validate the model in professional player cohorts from different leagues, different countries, and different age groups.

3. Lack of Methodological Clarity

Problem:The description of data preprocessing and feature engineering for the machine learning part is vague. For instance, how exactly were the "nonlinear and interaction terms" created? Which features were used? Similarly, the specific range and methods for hyperparameter tuning are not described, affecting the reproducibility of the study.

Suggestions:

(1) In the "Machine Learning Analysis" section, supplement the text with a table listing all features ultimately input into the model (e.g., weight, height, BMI, body surface area, and potentially constructed interaction terms like weight², weight × height).

(2) Provide a detailed description of the hyperparameter tuning process. For example, for the SVR model, specify the kernel function type (e.g., RBF kernel) and describe the range used for tuning parameters such as C, epsilon, and gamma using methods like GridSearch or RandomizedSearch, along with the final optimal values chosen. This would greatly enhance the scientific rigor of the study.

4. Contradictory Presentation and Over-interpretation of Results

Problem:The abstract mentions that the SVR model "reduced the error by 227 to 379 kcal/day." This value appears derived by subtracting the SVR's MAE from the "bias" of the traditional equations. This comparison is misleading. MAE measures the average absolute error at the individual level, while "bias" is the average difference (mean error). These are not the same concept, and directly subtracting them exaggerates the improvement. Furthermore, the SVR model's R² is only 0.169. Although this is better than the negative or near-zero values of the traditional equations, it still means the model can only explain approximately 17% of the variance in RMR, indicating its predictive capability is actually quite limited.

Suggestions:

(1) Rephrase the relevant descriptions in the abstract and conclusions. A more accurate statement would be: The SVR model's prediction error (MAE) was substantially lower than the average bias or RMSE of the traditional equations, demonstrating higher accuracy in individual prediction. Avoid directly subtracting values to derive a specific "reduction" range.

(2) In the discussion, provide a balanced interpretation of the R² value of 0.169. Acknowledge its progress compared to the traditional equations, while candidly pointing out that a large amount of unexplained variance remains. This highlights the complexity of RMR and suggests that future work may need to incorporate more predictive factors (such as training load, hormonal levels, more precise body composition data, etc.).

Competing Interests Statement is Vague

Problem:Some authors have affiliations with Breezing Co., the company that manufactures the device used in this study. Although the statement mentions "no financial compensation was received," "technical consulting and development support" in itself constitutes a potential conflict of interest that could influence the objectivity of the research. The statement claims this "did not influence the study outcomes," but this judgment should ultimately be made by the reviewers and readers.

Suggestion:

(1) It is recommended that the authors provide a clearer and more transparent competing interests statement. For example, they could specify the nature and duration of the consulting/support, and the measures taken to prevent bias (e.g., data analysis was performed by a third party not involved in the commercial collaboration). The current statement, "It did not influence the study outcomes," is overly subjective. PLOS has strict requirements regarding competing interests; vague language could potentially raise issues.

Minor Comments

Writing and Grammar: The overall readability of the manuscript is acceptable, but there are minor grammatical errors and instances of unnatural phrasing. For example, the sentence in the abstract, "corresponding to a reduction of 227 to 379 kcal·day-1 based on the difference between its MAE and the observed biases," as previously noted, is inaccurately phrased and grammatically awkward. It is suggested that the authors ask a native English-speaking colleague or a professional editing service to polish the language of the full manuscript to enhance fluency and professionalism.

Clarity of Figures/Tables:

Table 3:The title is in English, but the header for the first column is "Modelo" (Spanish/Portuguese). This should be unified to English, i.e., "Model." Please review the entire manuscript to ensure all elements are in English.

Figure 1:The figure legend mentions "RMSE and bias values," but these numerical values are not shown in the figure itself; only effect sizes are displayed. It is recommended to either annotate the RMSE and bias for key equations directly on the figure or provide this information in a separate table for more complete presentation.

Reference Format: Please check the reference format to ensure it fully complies with the journal requirements of PLOS One. For instance, in reference number 5, the journal name is written in all capitals; this should be corrected to the standard format.

Data Availability Statement: The authors state, "All relevant data are within the manuscript and its Supporting Information files." It is recommended that the authors upload the anonymized data for the 40 players used to train the SVR model (age, height, weight, IC-measured RMR values) as a Supporting Information file. This would genuinely make the data open, which is a core policy requirement of PLOS journals.

Additional Comments

Introduction Section The introduction effectively establishes the background and necessity of the study. It could be slightly streamlined by reducing general knowledge about energy expenditure in football and focusing more on the limitations of existing RMR prediction equations in athletic populations. This would better highlight the innovative aspects and urgency of this research.

Discussion Section: The discussion provides a thorough analysis of why the traditional equations fail. However, when discussing the advantages of the ML model, greater caution is needed to avoid giving the erroneous impression that the "ML model is good enough." Its "preliminary" nature and the need for "validation" must be repeatedly emphasized. Concurrently, future research directions could be made more concrete. For example, proposing an ideal study design: a prospective, multi-center cohort study collecting multi-dimensional data including DXA body composition, training load, and hormonal levels to build and validate more robust prediction models.

Conclusion

This is an interesting study with innovative potential. It highlights the significant inadequacy of traditional RMR prediction equations in elite athletes and explores the feasibility of machine learning as an alternative approach. However, the notable shortcomings concerning sample size, data age, methodological transparency, and result interpretation mean that, in its current form, it is not yet suitable for acceptance by PLOS One

If the authors are able to undertake a major revision, discussing the study's limitations candidly and in depth, providing more transparent methodological details, and offering a more rigorous and balanced interpretation of the results, then this paper could become highly valuable. The value of the revised manuscript would lie primarily in its role as a warning to practitioners against relying on traditional equations, and in providing important proof-of-concept and directional guidance for future research on exercise metabolism utilizing larger datasets and advanced methodologies. We encourage the authors to carefully revise the manuscript according to the comments above and look forward to seeing the revised version.

**********

6. PLOS authors have the option to publish the peer review history of their article (what does this mean?). If published, this will include your full peer review and any attached files.

If you choose “no”, your identity will remain anonymous but your review may still be made public.

**Do you want your identity to be public for this peer review?**  For information about this choice, including consent withdrawal, please see our Privacy Policy.

Reviewer #1: No

Reviewer #2: No

**********

[NOTE: If reviewer comments were submitted as an attachment file, they will be attached to this email and accessible via the submission site. Please log into your account, locate the manuscript record, and check for the action link "View Attachments". If this link does not appear, there are no attachment files.]

To ensure your figures meet our technical requirements, please review our figure guidelines: https://journals.plos.org/plosone/s/figures

You may also use PLOS’s free figure tool, NAAS, to help you prepare publication quality figures: https://journals.plos.org/plosone/s/figures#loc-tools-for-figure-preparation.

NAAS will assess whether your figures meet our technical requirements by comparing each figure against our figure specifications.

# Author comment: Author response to Decision Letter 1

**DOI:** 10.1371/journal.pone.0354973.r002

11 May 2026

Responses to Reviewer #1:

1.The machine learning sample consisted of 40 cases, which may be insufficient for constructing a robust predictive model (as acknowledged by the authors in the limitations section). A small sample size increases the risk of overfitting. Although the study conducted cross-validation and learning curve analyses, if feasible, it would be beneficial to report the effect size (as illustrated in Figure 1) of the predictive model to further validate model stability. In addition, while cross-validation was performed, the specific method should be clearly stated. Was k-fold cross-validation used, or another approach?

Answer: We thank the reviewer for this important observation. We acknowledge that the sample size (n = 40) is limited for developing complex predictive models; therefore, the present study is framed as a proof-of-concept.

To further support model stability, we calculated the effect size (Hedges’ g) for the optimized SVR model, obtaining a value of −0.05 (95% CI: −0.36 to 0.26), indicating no meaningful systematic bias relative to indirect calorimetry.

Additionally, we have clarified in the Methods section that the validation approach was based on Leave-One-Out Cross-Validation (LOOCV), which is particularly suitable for small datasets, as it maximizes data utilization while providing an almost unbiased estimate of model performance.

2.The model included body mass, stature, BMI, and BSA as predictors. Given that BMI and BSA are calculated from body mass and stature, correlations exist among these variables, potentially leading to multicollinearity. It is recommended that variance inflation factor (VIF) diagnostics be conducted to assess the extent of multicollinearity.

Answer: We thank the reviewer for this insightful comment. We agree that intrinsic correlations exist among body mass, stature, BMI, and body surface area, and VIF diagnostics confirmed elevated multicollinearity among these predictors.

However, the final model was based on Support Vector Regression (SVR) with a non-linear radial basis function (RBF) kernel, which is less sensitive to multicollinearity compared to traditional linear regression approaches. Specifically, SVR incorporates regularization (parameter C) and transforms the input space into a higher-dimensional feature space, allowing it to handle correlated predictors without compromising model stability or predictive performance.

To improve transparency, we have clarified this aspect in the Methods section and explicitly acknowledged the presence of multicollinearity and the rationale for using SVR as a robust alternative under these conditions.

3.In the study, the dataset was randomly partitioned into an 80–20 split for model training and external validation. However, this procedure still constitutes internal hold-out validation. True external validation would require testing the model on an independent dataset from a separate group of athletes.

Answer: We agree with the reviewer; this point has been clarified in the discussion.

4.The discussion section, the authors may consider discussing whether similar modeling approaches have been adopted in prior research and comparing their findings with existing studies. This would help clarify the theoretical contribution and practical implications of the present study.

Answer: We thank the reviewer for this suggestion. To the best of our knowledge, there are currently no studies applying a comparable machine-learning framework for RMR prediction specifically in professional soccer players. This has now been clarified in the Discussion section, where we also position our findings within the broader context of emerging data-driven approaches in sports science.

Responses to Reviewer #2:

1.Small Sample Size and Outdated Data (Critical Limitation)

Problem: The study sample consists of only 40 individuals, with data collected in 2014 (referred to as the "Opening Tournament 2014"). This small sample size not only limits the training efficacy and generalizability of the machine learning model (even though the authors use learning curves to suggest no overfitting, the small sample remains a major constraint) but also raises questions about whether data nearly a decade old accurately reflects the physiological characteristics of current professional football players. Training methodologies, nutritional strategies, and athlete body composition have evolved significantly over the past decade.

Suggestions:

(1)In the discussion section, this must be addressed more profoundly and transparently as the primary limitation. Clearly state the impact of the small sample size and outdated data on the generalizability of the conclusions.

Answer: We thank the reviewer for this important observation. We have revised the Discussion section to explicitly acknowledge the small sample size and the use of data collected in 2014 as critical limitations. Their potential impact on generalizability and contemporary relevance is now discussed in greater depth.

(2)Explicitly frame the machine learning model as a "pilot exploration," as already indicated in the title and text. Emphasize that its results require external validation in larger, multi-center, and more recent cohorts before any practical application can be considered.

Answer: We agree with the reviewer. The manuscript has been revised to clearly state that the machine-learning model should be considered exploratory. We now emphasize that external validation in independent and multicenter cohorts is required before any practical application.

(3)If possible, attempt to contact the authors or the club to see if newer or additional data can be obtained to augment the sample. However, this is often difficult to achieve, so a frank acknowledgment of the limitations is the more realistic path forward.

Answer: We appreciate the reviewer’s suggestion. Unfortunately, it was not possible to obtain additional or more recent data from the club or the original data sources. As recommended, we have explicitly acknowledged this limitation in the revised manuscript and discussed its implications for the generalizability and contemporary relevance of the findings.

2.Lack of External Validation for the Machine Learning Model

Problem:The model's superior performance is demonstrated solely based on an internal validation set (20% of the data) and cross-validation. In machine learning research, the absence of an independent external validation set is a critical weakness. This leaves the true generalizability of the model unknown, with a risk of overfitting to the specific characteristics of these 40 players. Although the authors examined learning curves, this does not substitute for external validation.

Suggestions:

(1) Explicit Differentiation:In the title, abstract, and conclusions, employ more cautious phrasing, such as "preliminary results suggest," "demonstrated strong performance in internal validation," etc., to avoid creating the impression that the model is ready for widespread application.

Asnwer: We thank the reviewer for this important suggestion. The title, abstract, and conclusions have been revised to adopt a more cautious and accurate tone. Specifically, we have introduced terms such as “preliminary” and clarified that the machine learning model demonstrated improved performance under internal validation conditions. We have also emphasized the exploratory nature of the findings and the need for external validation before practical application.

(2) Future Research Direction:In the discussion section, emphasize "external validation" as the most critical next step for research. Concrete suggestions could be made that future studies should validate the model in professional player cohorts from different leagues, different countries, and different age groups.

Answer: We agree with the reviewer. The manuscript has been revised to clearly state that the machine-learning model should be considered exploratory. We now emphasize that external validation in independent and multicenter cohorts is required before any practical application.

3.Lack of Methodological Clarity

Problem:The description of data preprocessing and feature engineering for the machine learning part is vague. For instance, how exactly were the "nonlinear and interaction terms" created? Which features were used? Similarly, the specific range and methods for hyperparameter tuning are not described, affecting the reproducibility of the study.

Suggestions:

(1) In the "Machine Learning Analysis" section, supplement the text with a table listing all features ultimately input into the model (e.g., weight, height, BMI, body surface area, and potentially constructed interaction terms like weight², weight × height).

Answer: We thank the reviewer for this important observation. We have substantially improved the transparency and reproducibility of the machine learning methodology. Specifically, we expanded the Data Preprocessing section to explicitly describe the feature engineering process, including the generation of nonlinear (e.g., squared terms) and interaction terms (e.g., body mass × stature).

In addition, we have incorporated a new table (Table X) in the Machine Learning Analysis section that details all input features used in the model, including primary (e.g., body mass, stretch stature, age), derived (BMI, BSA), nonlinear, and interaction variables, along with their formulas and rationale.

These additions provide a clear and reproducible description of the predictors included in the modeling process.

(2) Provide a detailed description of the hyperparameter tuning process. For example, for the SVR model, specify the kernel function type (e.g., RBF kernel) and describe the range used for tuning parameters such as C, epsilon, and gamma using methods like GridSearch or RandomizedSearch, along with the final optimal values chosen. This would greatly enhance the scientific rigor of the study.

Answer: We appreciate the reviewer’s suggestion to improve methodological transparency. In response, we have added a dedicated subsection (Hyperparameter Optimization of the SVR Model) within the Machine Learning Analysis section.

In this subsection, we now explicitly describe the optimization procedure, including the use of GridSearchCV with 5-fold cross-validation, the selection of a radial basis function (RBF) kernel, and the explored parameter ranges for C, ε, and γ. Hyperparameter tuning was performed using 5-fold cross-validation, while final model validation was conducted using Leave-One-Out Cross-Validation (LOOCV) to maximize data utilization given the small sample size. We also report the final optimal values selected (C = 10, ε = 0.1, γ = ‘scale’).

4.Contradictory Presentation and Over-interpretation of Results

Problem:The abstract mentions that the SVR model "reduced the error by 227 to 379 kcal/day." This value appears derived by subtracting the SVR's MAE from the "bias" of the traditional equations. This comparison is misleading. MAE measures the average absolute error at the individual level, while "bias" is the average difference (mean error). These are not the same concept, and directly subtracting them exaggerates the improvement. Furthermore, the SVR model's R² is only 0.169. Although this is better than the negative or near-zero values of the traditional equations, it still means the model can only explain approximately 17% of the variance in RMR, indicating its predictive capability is actually quite limited.

Answer: We agree with the reviewer. The conclusions have been revised to avoid overinterpretation of the machine learning model. We now explicitly state that the findings are preliminary and exploratory, and that the model should be considered a proof of concept rather than a ready-to-use tool. We have also highlighted the need for cautious interpretation and further validation in larger and independent cohorts.

Suggestions:

(1) Rephrase the relevant descriptions in the abstract and conclusions. A more accurate statement would be: The SVR model's prediction error (MAE) was substantially lower than the average bias or RMSE of the traditional equations, demonstrating higher accuracy in individual prediction. Avoid directly subtracting values to derive a specific "reduction" range.

Answer: We thank the reviewer for identifying this important issue. The misleading comparison between MAE and bias has been removed from the abstract and conclusions. We have reformulated the results to provide a more appropriate interpretation, stating that the MAE of the SVR model was lower than the RMSE and average bias observed in traditional equations, without directly subtracting these metrics.

(2) In the discussion, provide a balanced interpretation of the R² value of 0.169. Acknowledge its progress compared to the traditional equations, while candidly pointing out that a large amount of unexplained variance remains. This highlights the complexity of RMR and suggests that future work may need to incorporate more predictive factors (such as training load, hormonal levels, more precise body composition data, etc.).

Answer: We agree with the reviewer and have revised the manuscript accordingly. The abstract and conclusions now explicitly acknowledge that the SVR model explains a limited proportion of the variance (R² = 0.169). We have incorporated a more balanced interpretation, emphasizing both the relative improvement over traditional equations and the remaining unexplained variability.

5.Competing Interests Statement is Vague

Problem:Some authors have affiliations with Breezing Co., the company that manufactures the device used in this study. Although the statement mentions "no financial compensation was received," "technical consulting and development support" in itself constitutes a potential conflict of interest that could influence the objectivity of the research. The statement claims this "did not influence the study outcomes," but this judgment should ultimately be made by the reviewers and readers.

Suggestion:

(1) It is recommended that the authors provide a clearer and more transparent competing interests statement. For example, they could specify the nature and duration of the consulting/support, and the measures taken to prevent bias (e.g., data analysis was performed by a third party not involved in the commercial collaboration). The current statement, "It did not influence the study outcomes," is overly subjective. PLOS has strict requirements regarding competing interests; vague language could potentially raise issues.

Answer: We thank the reviewer for this important comment. The Conflict of Interest statement has been revised to improve transparency and clarity. Specifically, we have provided a more precise description of the nature of the collaboration and removed subjective statements. Additionally, we have clarified that the statistical analyses were conducted by an author independent of this collaboration to minimize potential bias.

Minor Comments

Writing and Grammar: The overall readability of the manuscript is acceptable, but there are minor grammatical errors and instances of unnatural phrasing. For example, the sentence in the abstract, "corresponding to a reduction of 227 to 379 kcal·day-1 based on the difference between its MAE and the observed biases," as previously noted, is inaccurately phrased and grammatically awkward. It is suggested that the authors ask a native English-speaking colleague or a professional editing service to polish the language of the full manuscript to enhance fluency and professionalism.

Clarity of Figures/Tables:

Table 3:The title is in English, but the header for the first column is "Modelo" (Spanish/Portuguese). This should be unified to English, i.e., "Model." Please review the entire manuscript to ensure all elements are in English.

Answer: Corrected. The term has been revised to “Model”, and the manuscript has been carefully checked to ensure consistency in the use of English throughout.

Figure 1:The figure legend mentions "RMSE and bias values," but these numerical values are not shown in the figure itself; only effect sizes are displayed. It is recommended to either annotate the RMSE and bias fo

Attachment Submitted filename: 1. Response to Reviewers.docx

# Peer review: Decision Letter 1

**DOI:** 10.1371/journal.pone.0354973.r003

**Authors:** Zulkarnain Jaafar

2 Jun 2026

PONE-D-26-02771R1

Estimation of Resting Metabolic Rate in Professional Soccer Players: A Cross-Sectional Study Comparing Traditional Predictive Equations and a Preliminary Machine Learning Model Against Indirect Calorimetry

PLOS One

Dear Dr. López-Gil,

Thank you for submitting your manuscript to PLOS ONE. After careful consideration, we feel that it has merit but does not fully meet PLOS ONE’s publication criteria as it currently stands. Therefore, we invite you to submit a revised version of the manuscript that addresses the points raised during the review process.

Please submit your revised manuscript by Jul 17 2026 11:59PM. If you will need more time than this to complete your revisions, please reply to this message or contact the journal office at plosone@plos.org. When you're ready to submit your revision, log on to https://www.editorialmanager.com/pone/ and select the 'Submissions Needing Revision' folder to locate your manuscript file.

Please include the following items when submitting your revised manuscript:

- A letter that responds to each point raised by the academic editor and reviewer(s). You should upload this letter as a separate file labeled 'Response to Reviewers'.
- A marked-up copy of your manuscript that highlights changes made to the original version. You should upload this as a separate file labeled 'Revised Manuscript with Track Changes'.
- An unmarked version of your revised paper without tracked changes. You should upload this as a separate file labeled 'Manuscript'.

If you would like to make changes to your financial disclosure, please include your updated statement in your cover letter. Guidelines for resubmitting your figure files are available below the reviewer comments at the end of this letter.

If applicable, we recommend that you deposit your laboratory protocols in protocols.io to enhance the reproducibility of your results. Protocols.io assigns your protocol its own identifier (DOI) so that it can be cited independently in the future. For instructions see: https://journals.plos.org/plosone/s/submission-guidelines#loc-laboratory-protocols. Additionally, PLOS ONE offers an option for publishing peer-reviewed Lab Protocol articles, which describe protocols hosted on protocols.io. Read more information on sharing protocols at https://plos.org/protocols?utm_medium=editorial-email&utm_source=authorletters&utm_campaign=protocols.

As the corresponding author, your ORCID iD is verified in the submission system and will appear in the published article. PLOS supports the use of ORCID, and we encourage all coauthors to register for an ORCID iD and use it as well. Please encourage your coauthors to verify their ORCID iD within the submission system before final acceptance, as unverified ORCID iDs will not appear in the published article. *Only*  the individual author can complete the verification step; PLOS staff *cannot*  verify ORCID iDs on behalf of authors.

We look forward to receiving your revised manuscript.

Kind regards,

Zulkarnain Jaafar

Academic Editor

PLOS One

Journal Requirements:

If the reviewer comments include a recommendation to cite specific previously published works, please review and evaluate these publications to determine whether they are relevant and should be cited. There is no requirement to cite these works unless the editor has indicated otherwise.

Additional Editor Comments:

Dear Author, your manuscript still requiring major corrections. Please make necessary changes based on the comments provided by the reviewer.

[Note: HTML markup is below. Please do not edit.]

Reviewers' comments:

Reviewer's Responses to Questions

**Comments to the Author**

1. If the authors have adequately addressed your comments raised in a previous round of review and you feel that this manuscript is now acceptable for publication, you may indicate that here to bypass the “Comments to the Author” section, enter your conflict of interest statement in the “Confidential to Editor” section, and submit your "Accept" recommendation.

Reviewer #1: All comments have been addressed

Reviewer #2: (No Response)

**********

2. Is the manuscript technically sound, and do the data support the conclusions?

The manuscript must describe a technically sound piece of scientific research with data that supports the conclusions. Experiments must have been conducted rigorously, with appropriate controls, replication, and sample sizes. The conclusions must be drawn appropriately based on the data presented.

Reviewer #1: Yes

Reviewer #2: No

**********

3. Has the statistical analysis been performed appropriately and rigorously?

Reviewer #1: Yes

Reviewer #2: No

**********

4. Have the authors made all data underlying the findings in their manuscript fully available?

The PLOS Data policy requires authors to make all data underlying the findings described in their manuscript fully available without restriction, with rare exception (please refer to the Data Availability Statement in the manuscript PDF file). The data should be provided as part of the manuscript or its supporting information, or deposited to a public repository. For example, in addition to summary statistics, the data points behind means, medians and variance measures should be available. If there are restrictions on publicly sharing data—e.g. participant privacy or use of data from a third party—those must be specified.

Reviewer #1: Yes

Reviewer #2: No

**********

5. Is the manuscript presented in an intelligible fashion and written in standard English?

PLOS ONE does not copyedit accepted manuscripts, so the language in submitted articles must be clear, correct, and unambiguous. Any typographical or grammatical errors should be corrected at revision, so please note any specific errors here.

Reviewer #1: Yes

Reviewer #2: No

**********

6. Review Comments to the Author

Please use the space provided to explain your answers to the questions above. You may also include additional comments for the author, including concerns about dual publication, research ethics, or publication ethics. (Please upload your review as an attachment if it exceeds 20,000 characters)

Reviewer #1: The sample size is the main concern of this study; the authors have acknowledged this point in the limitations section and clarified that this study is intended as a proof-of-concept.

Reviewer #2: (No Response)

**********

7. PLOS authors have the option to publish the peer review history of their article (what does this mean?). If published, this will include your full peer review and any attached files.

If you choose “no”, your identity will remain anonymous but your review may still be made public.

**Do you want your identity to be public for this peer review?**  For information about this choice, including consent withdrawal, please see our Privacy Policy.

Reviewer #1: No

Reviewer #2: No

**********

[NOTE: If reviewer comments were submitted as an attachment file, they will be attached to this email and accessible via the submission site. Please log into your account, locate the manuscript record, and check for the action link "View Attachments". If this link does not appear, there are no attachment files.]

To ensure your figures meet our technical requirements, please review our figure guidelines: https://journals.plos.org/plosone/s/figures

You may also use PLOS’s free figure tool, NAAS, to help you prepare publication quality figures: https://journals.plos.org/plosone/s/figures#loc-tools-for-figure-preparation.

NAAS will assess whether your figures meet our technical requirements by comparing each figure against our figure specifications.

Attachment Submitted filename: Minor Revision .doc

# Author comment: Author response to Decision Letter 2

**DOI:** 10.1371/journal.pone.0354973.r004

4 Jun 2026

Response to Reviewer #1

Manuscript: Optimizing Football Analytics: Dimensionality Reduction Meets Machine Learning for Offensive and Defensive Player Profiling

PLOS ONE — PONE-D-25-61870R1

We thank the reviewer for the thorough and constructive second round of comments. Below we provide a point-by-point response to each remaining concern. All revised text is highlighted in the tracked-changes version of the manuscript. Recalculated figures are based on the processed dataset (2,689 records; 124 source variables; 2,530 unique players) using a leakage-controlled workflow in which the train/test partition is performed first and imputation, scaling, and PCA are fitted exclusively on training data.

────────────────────────────────────────────────────────────────────────────────

Point 1: PCA Target–Predictor Circularity

Reviewer comment:

The SHAP results show that the top predictors of PC1_Offensive (Goals, SoT, Shots, RecProg, G/Sh) are the same variables that load most heavily on PC1_Offensive itself, confirming circularity. The reviewer requested: (i) explicit variable lists for PCA construction versus regression predictors; (ii) justification of any overlap; and (iii) confirmation that PCA, scaling, and imputation were fitted on training data only. A sensitivity analysis excluding overlapping variables was also requested.

Response:

We fully acknowledge the circularity issue raised and have implemented a complete separation between the PCA-construction variables and the regression predictor set. An automated audit confirms zero overlap.

(i) Explicit variable lists:

Offensive PCA variables: Goals_p90, Shots_p90, SoT_p90, G/Sh, G/SoT, Assists_p90, PasAss_p90, PPA_p90, CrsPA_p90, PasProg_p90, SCA_p90, GCA_p90, CarProg_p90, RecProg_p90.

Defensive PCA variables: Tkl_p90, Int_p90, Blocks_p90, Clr_p90, Tkl+Int_p90, TklW_p90, TklDef3rd, TklMid3rd, TklAtt3rd.

Regression predictors: All remaining numeric and contextual variables (position, league, age, minutes, passing, carrying, aerial, touch-location, and discipline metrics), explicitly excluding every PCA-construction variable and its base/per-90 equivalents.

(ii) Justification of overlap:

The originally submitted predictor set retained the index-defining metrics, producing the circularity identified by the reviewer. This overlap has been completely removed; an automated check aborts execution if any overlap is detected.

(iii) Train-only fitting + sensitivity analysis:

Median imputation, StandardScaler, and PCA are now fitted exclusively on the training partition and applied unchanged to the test set. A sensitivity analysis with two exclusion levels (exact variable match and domain-level exclusion) confirmed that performance and SHAP rankings are not driven by index-defining variables. The revised SHAP rankings are shown below:

Target

SHAP (original — circular)

SHAP (revised — non-circular)

PC1_Offensive

Goals, SoT, Shots, RecProg, G/Sh

TouAtt3rd, ScaPassLive, GcaPassLive, TouAttPen, CPA

PC1_Defensive

TklMid3rd, Tkl+Int, Tkl

TklWon, TklDri, BlkPass, BlkSh, Recov

After removing the overlap, the most influential predictors are involvement and recovery metrics that do not form part of the index definitions, resolving the circularity concern. Figures 3–6 in the revised manuscript show the updated, non-circular SHAP visualizations.

Manuscript change: The paragraph "Non-circularity and leakage control" has been added to the Methods section with the full variable lists, audit confirmation, and explicit statement that all data-dependent steps were fitted on training data only.

────────────────────────────────────────────────────────────────────────────────

Point 2: Missing-Data Handling

Reviewer comment:

The new imputation description (Item 6) is acceptable, but the original generic paragraph (Item 3) was left in place and now contradicts it. The reviewer requested removal of Item 3 and reporting of overall missingness extent, confirming that imputation was fitted on training data only.

Response:

The generic Item 3 paragraph has been deleted from the manuscript. The note currently visible in the Methods section confirms this removal: "[Item removed per reviewer request: this generic description contradicted the detailed missing-data workflow described below and has been deleted.]"

Regarding the extent of missingness: overall predictor missingness in the processed dataset was 0.0%, as count-based statistics with structural zeros were coded as zero at source. The structured imputation workflow (median for numeric variables; explicit 'Unknown' category for categorical variables) was fitted exclusively on the training partition.

Manuscript change: Item 3 removed; missingness rate (0.0%) and train-only imputation fitting explicitly stated in the Methods section.

────────────────────────────────────────────────────────────────────────────────

Point 3: Train/Test Split — Player-Grouped Analysis and Hyperparameter Tuning

Reviewer comment:

The concern about player-grouped splitting remained unaddressed analytically. If the dataset contains repeated player observations, the random 80/20 split may leak the same player into train and test, inflating R². The reviewer requested either a player-grouped reanalysis or a sensitivity analysis comparing both. Hyperparameter tuning protocol also needed description.

Response:

The dataset contains 2,689 records for 2,530 unique players (159 repeated observations). We re-executed the full analysis using a player-grouped 80/20 split (verified zero player overlap between partitions) and included a random split as a sensitivity analysis:

Target

Random split (R²)

Player-grouped split (R²)

Difference

PC1_Offensive

0.896

0.913

+0.017

PC1_Defensive

0.905

0.927

+0.022

The player-grouped split produced comparable (marginally higher) R² values relative to the random split, indicating that repeated players did not inflate model performance. All results reported in the revised manuscript correspond to the player-grouped analysis.

Hyperparameter protocol: Hyperparameters were fixed and pre-specified without automated search: tree models — max_depth = 10, min_samples_split = 10; Random Forest — n_estimators = 200; random seed = 42; linear models — default regularization. This is now explicitly stated in the Methods section.

Manuscript change: Predictive Modeling subsection updated with player-grouped split description, sensitivity analysis results, and full hyperparameter specification.

────────────────────────────────────────────────────────────────────────────────

Point 4: Data Availability

Reviewer comment:

Three different and contradictory data availability statements existed across the submission form, the manuscript, and page 8. PLOS ONE does not accept 'available on request.' The reviewer requested deposit of the processed dataset and analysis code in a public repository with a DOI, or formal justification of an exemption.

Response:

We thank the reviewer for this important correction. All 'available on request' statements have been removed from the manuscript. The source data underlying this study are publicly accessible and can be downloaded directly from Kaggle:

Dataset: https://www.kaggle.com/datasets/vivovinco/20222023-football-player-stats?select=2022-2023+Football+Player+Stats.csv

The processed analytical dataset was derived from this publicly accessible football performance platform (2022–2023 Football Player Stats). The complete analysis code (preprocessing, PCA construction, all eight supervised models, the leakage-controlled player-grouped split, and SHAP analyses) will additionally be deposited in a public repository (Zenodo or OSF) with a citable DOI prior to final acceptance.

The Data Availability Statement in the revised manuscript now reads: "The processed analytical dataset was obtained from a publicly accessible platform and can be downloaded from: https://www.kaggle.com/datasets/vivovinco/20222023-football-player-stats?select=2022-2023+Football+Player+Stats.csv. The full analysis code is openly available at [repository DOI to be inserted upon deposit]."

Manuscript change: Data Availability Statement updated with the direct Kaggle URL. 'Available on request' language removed from all three locations.

────────────────────────────────────────────────────────────────────────────────

Point 5: Language Standardization — Figure 2 Legend

Reviewer comment:

The body text is now in English; however, Figure 2's legend still contained 'Posición / Minutos' in Spanish. The reviewer requested re-export of the figure.

Response:

Figure 2 has been re-exported with the axis label and legend fully in English: 'Position / Minutes' replaces 'Posición / Minutos'. The updated figure file meets PLOS ONE resolution requirements (≥300 DPI, TIFF or EPS format).

Manuscript change: Figure 2 replaced with English-only version. All figure legends and axis labels verified to be in English throughout the manuscript.

────────────────────────────────────────────────────────────────────────────────

Point 6: Table 1 — 'Total' Column Definition

Reviewer comment:

The 'Total' column remained undefined and its values equaled the Offensive column in every row, suggesting duplication. The reviewer requested a table footnote and Methods definition of 'Total'.

Response:

The 'Total' column has been removed from Table 1. As the revised table footnote states: "The previously included 'Total' column was undefined and merely duplicated the Offensive values; it has been removed and no aggregate score is defined." The table now reports Offensive (PC1_Offensive) and Defensive (PC1_Defensive) performance separately, with updated values from the leakage-controlled, player-grouped analysis:

Model

Defensive R²

Def. MAE

Def. RMSE

Offensive R²

Off. MAE

Off. RMSE

Random Forest

0.927

0.421

0.642

0.913

0.526

0.781

SVR

0.511

0.939

1.726

0.615

1.034

2.164

Ridge

0.502

0.967

1.757

0.614

1.096

2.166

Linear Regression

0.501

0.967

1.762

0.611

1.104

2.184

Decision Tree

0.393

1.089

2.142

0.504

1.157

2.785

K Neighbors

0.431

1.041

2.008

0.576

1.094

2.381

ElasticNet

0.103

1.328

3.168

0.189

1.574

4.555

Lasso

−0.002

1.408

3.541

0.066

1.699

5.248

Random Forest was retained as the reference model for SHAP interpretation because it achieved the strongest performance among non-linear ensemble models capable of capturing interaction effects (offensive R² = 0.913; defensive R² = 0.927). Regularized linear models (Ridge) reached marginally higher R² (0.933 and 0.942) but do not support non-linear SHAP decomposition; Random Forest is therefore presented as the selected model on interpretability grounds, not as the sole highest-R² algorithm.

Manuscript change: 'Total' column removed; Table 1 footnote added; best-model rationale clarified in both the Results and Methods sections.

────────────────────────────────────────────────────────────────────────────────

Summary of Changes

We believe the manuscript now fully addresses all six points raised by Reviewer #1. The key changes are: (1) complete PCA/predictor separation with automated overlap audit and updated non-circular SHAP figures; (2) removal of the contradictory generic imputation paragraph; (3) player-grouped train/test split with sensitivity analysis and explicit hyperparameter specification; (4) unified data availability statement with direct Kaggle URL for the source dataset and repository DOI for analysis code; (5) re-exported Figure 2 with English-only legend; and (6) removal of the undefined 'Total' column with revised Table 1 footnote.

We appreciate the reviewer's diligence in identifying these issues, which have substantively improved the methodological transparency and reproducibility of the study.

On behalf of all authors,

Dr. José Francisco López-Gil

Corresponding Author — PONE-D-25-61870R1

Attachment Submitted filename: Response to reviewers_R2 (PlosOne).docx

# Peer review: Decision Letter 2

**DOI:** 10.1371/journal.pone.0354973.r005

**Authors:** Zulkarnain Jaafar

22 Jun 2026

PONE-D-26-02771R2

Estimation of Resting Metabolic Rate in Professional Soccer Players: A Cross-Sectional Study Comparing Traditional Predictive Equations and a Preliminary Machine Learning Model Against Indirect Calorimetry

PLOS One

Dear Dr.  López-Gil,

Thank you for submitting your manuscript to PLOS ONE. After careful consideration, we feel that it has merit but does not fully meet PLOS ONE’s publication criteria as it currently stands. Therefore, we invite you to submit a revised version of the manuscript that addresses the points raised during the review process.

Please submit your revised manuscript by Aug 06 2026 11:59PM. If you will need more time than this to complete your revisions, please reply to this message or contact the journal office at plosone@plos.org. When you're ready to submit your revision, log on to https://www.editorialmanager.com/pone/ and select the 'Submissions Needing Revision' folder to locate your manuscript file.

Please include the following items when submitting your revised manuscript:

- A letter that responds to each point raised by the academic editor and reviewer(s). You should upload this letter as a separate file labeled 'Response to Reviewers'.
- A marked-up copy of your manuscript that highlights changes made to the original version. You should upload this as a separate file labeled 'Revised Manuscript with Track Changes'.
- An unmarked version of your revised paper without tracked changes. You should upload this as a separate file labeled 'Manuscript'.

If you would like to make changes to your financial disclosure, please include your updated statement in your cover letter. Guidelines for resubmitting your figure files are available below the reviewer comments at the end of this letter.

If applicable, we recommend that you deposit your laboratory protocols in protocols.io to enhance the reproducibility of your results. Protocols.io assigns your protocol its own identifier (DOI) so that it can be cited independently in the future. For instructions see: https://journals.plos.org/plosone/s/submission-guidelines#loc-laboratory-protocols. Additionally, PLOS ONE offers an option for publishing peer-reviewed Lab Protocol articles, which describe protocols hosted on protocols.io. Read more information on sharing protocols at https://plos.org/protocols?utm_medium=editorial-email&utm_source=authorletters&utm_campaign=protocols.

As the corresponding author, your ORCID iD is verified in the submission system and will appear in the published article. PLOS supports the use of ORCID, and we encourage all coauthors to register for an ORCID iD and use it as well. Please encourage your coauthors to verify their ORCID iD within the submission system before final acceptance, as unverified ORCID iDs will not appear in the published article. *Only*  the individual author can complete the verification step; PLOS staff *cannot*  verify ORCID iDs on behalf of authors.

We look forward to receiving your revised manuscript.

Kind regards,

Zulkarnain Jaafar

Academic Editor

PLOS One

Journal Requirements:

If the reviewer comments include a recommendation to cite specific previously published works, please review and evaluate these publications to determine whether they are relevant and should be cited. There is no requirement to cite these works unless the editor has indicated otherwise.

Additional Editor Comments:

Dear Author, please revise your manuscript based on the comments provided by the reviewer.

[Note: HTML markup is below. Please do not edit.]

Reviewers' comments:

Reviewer's Responses to Questions

**Comments to the Author**

1. If the authors have adequately addressed your comments raised in a previous round of review and you feel that this manuscript is now acceptable for publication, you may indicate that here to bypass the “Comments to the Author” section, enter your conflict of interest statement in the “Confidential to Editor” section, and submit your "Accept" recommendation.

Reviewer #2: (No Response)

**********

2. Is the manuscript technically sound, and do the data support the conclusions?

The manuscript must describe a technically sound piece of scientific research with data that supports the conclusions. Experiments must have been conducted rigorously, with appropriate controls, replication, and sample sizes. The conclusions must be drawn appropriately based on the data presented.

Reviewer #2: Partly

**********

3. Has the statistical analysis been performed appropriately and rigorously?

Reviewer #2: No

**********

4. Have the authors made all data underlying the findings in their manuscript fully available?

The PLOS Data policy requires authors to make all data underlying the findings described in their manuscript fully available without restriction, with rare exception (please refer to the Data Availability Statement in the manuscript PDF file). The data should be provided as part of the manuscript or its supporting information, or deposited to a public repository. For example, in addition to summary statistics, the data points behind means, medians and variance measures should be available. If there are restrictions on publicly sharing data—e.g. participant privacy or use of data from a third party—those must be specified.

Reviewer #2: No

**********

5. Is the manuscript presented in an intelligible fashion and written in standard English?

PLOS ONE does not copyedit accepted manuscripts, so the language in submitted articles must be clear, correct, and unambiguous. Any typographical or grammatical errors should be corrected at revision, so please note any specific errors here.

Reviewer #2: Yes

**********

6. Review Comments to the Author

Please use the space provided to explain your answers to the questions above. You may also include additional comments for the author, including concerns about dual publication, research ethics, or publication ethics. (Please upload your review as an attachment if it exceeds 20,000 characters)

Reviewer #2: Reviewer Comments

Recommendation: Major Revision

This revised manuscript addresses an applied and relevant question: whether commonly used resting metabolic rate prediction equations are suitable for professional soccer players, using indirect calorimetry as the reference method, and whether a preliminary machine-learning model may improve prediction. The topic is of practical interest to sports nutrition and athlete monitoring, and the comparison against indirect calorimetry is appropriate. The manuscript has also improved in tone by presenting the machine-learning component more cautiously as exploratory.

However, several issues still require clarification before the manuscript can be considered suitable for publication. The main concerns relate to the consistency of the submitted files, the strength of the claims regarding the SVR model, the small sample size, model validation, multicollinearity among predictors, and the need for full transparency regarding ethics, data availability, and numerical consistency.

Major Comments

1. Potential inconsistency in the submitted review file

The reviewer PDF appears to contain a response-to-reviewers section referring to a different manuscript, titled “Optimizing Football Analytics: Dimensionality Reduction Meets Machine Learning for Offensive and Defensive Player Profiling,” with comments about PCA, SHAP, offensive and defensive player profiling, and train-test leakage. This content does not correspond to the present manuscript on resting metabolic rate in professional soccer players.

The authors and editorial office should verify whether this is a file-compilation error or whether incorrect response material was included in the submission. A clean and internally consistent manuscript package should be submitted.

2. The machine-learning model should remain clearly framed as exploratory

The SVR model is potentially interesting, but the dataset is small, with only 40 participants. If the model development used an 80/20 split, the held-out validation subset would include only approximately eight participants, making performance estimates highly unstable. Although the model showed lower MAE/RMSE than traditional equations, the reported R² of 0.169 indicates that most between-individual variability in RMR remains unexplained.

The manuscript should avoid any wording suggesting that the SVR model is validated, generalizable, reliable for practice, or ready for applied use. It should consistently be described as a preliminary, proof-of-concept internal-validation analysis requiring external validation.

3. Validation strategy and data leakage prevention must be fully transparent

The Methods should clearly state the exact validation procedure, including:

o the training/validation split ratio;

o whether hyperparameter tuning was conducted only within the training subset;

o whether preprocessing, outlier screening, scaling, and feature engineering were fitted only on the training data;

o whether the held-out subset was used only once for final internal validation.

Given the small sample, the authors should also explicitly acknowledge the risk of optimistic performance estimates.

4. Multicollinearity among predictors should be handled cautiously

Several predictors used in the SVR model are mathematically related, including body mass, stature, BMI, BSA, squared body mass, and body mass × stature. This creates strong multicollinearity by construction, especially problematic with n = 40.

The authors should report VIF diagnostics or clearly state that the model is intended only for prediction and that no inference is made regarding the independent contribution or biological importance of individual predictors.

5. Indirect calorimetry procedures require sufficient methodological detail

Since indirect calorimetry is treated as the reference standard, the manuscript should provide adequate detail on the measurement protocol, including device calibration, fasting status, prior exercise restrictions, caffeine or stimulant control, environmental conditions, resting period, measurement duration, steady-state criteria, and handling of implausible respiratory exchange ratio values. Without these details, the validity of the reference RMR values is difficult to assess.

6. Numerical consistency across text, tables, and figures should be checked

The response indicates that previous inconsistencies existed regarding relative error rankings, bias ranges, Pearson correlations, and the definition of “best-performing” equations. The revised manuscript should ensure that all numerical statements in the Abstract, Results, Discussion, tables, and figure captions are fully consistent.

In particular, “best-performing” should be defined consistently, as different metrics may identify different equations: lowest RMSE, lowest bias, highest ICC/CCC, or lowest relative error.

7. Ethics and consent timeline should be clarified

The manuscript should clearly explain the apparent discrepancy between data collection in 2014 and the later ethics approval code. If the approval covered retrospective analysis and anonymized data sharing, this should be explicitly stated. The authors should also confirm that informed consent, and parental consent for minors where applicable, covered research use and public sharing of anonymized data.

Minor Comments

1. The conclusion “traditional equations are not suitable” may be too strong. A more cautious phrasing would be “traditional equations showed poor agreement in this sample of professional soccer players.”

2. Units should be standardized throughout the manuscript, especially kcal/day versus kcal·day⁻¹.

3. The abstract should maintain the cautious framing of the SVR model and avoid implying external validity.

4. The competing-interest statement should clearly identify any relationship with the indirect-calorimetry device or company, the authors involved, the role of the company, and who conducted the statistical analyses.

5. The Data Availability Statement should provide a clear and accessible link to the dataset and specify whether the shared dataset is raw, processed, or anonymized.

Overall Assessment

The manuscript has potential and addresses a useful applied problem. The strongest contribution is the finding that traditional RMR prediction equations show poor agreement with indirect calorimetry in this specific population. The machine-learning component is interesting but should remain secondary and exploratory because of the small sample size, limited internal validation, low R², and absence of external validation.

I recommend major revision, primarily to ensure file consistency, strengthen methodological transparency, moderate the interpretation of the SVR model, and confirm that all numerical and ethical statements are internally consistent.

**********

7. PLOS authors have the option to publish the peer review history of their article (what does this mean?). If published, this will include your full peer review and any attached files.

If you choose “no”, your identity will remain anonymous but your review may still be made public.

**Do you want your identity to be public for this peer review?**  For information about this choice, including consent withdrawal, please see our Privacy Policy.

Reviewer #2: No

**********

[NOTE: If reviewer comments were submitted as an attachment file, they will be attached to this email and accessible via the submission site. Please log into your account, locate the manuscript record, and check for the action link "View Attachments". If this link does not appear, there are no attachment files.]

To ensure your figures meet our technical requirements, please review our figure guidelines: https://journals.plos.org/plosone/s/figures

You may also use PLOS’s free figure tool, NAAS, to help you prepare publication quality figures: https://journals.plos.org/plosone/s/figures#loc-tools-for-figure-preparation.

NAAS will assess whether your figures meet our technical requirements by comparing each figure against our figure specifications.

# Author comment: Author response to Decision Letter 3

**DOI:** 10.1371/journal.pone.0354973.r006

26 Jun 2026

Response to Reviewers

Manuscript ID: PON-D-26-02771R2

Title: Estimation of basal metabolic rate in professional soccer players: a cross-sectional study comparing traditional predictive equations and a preliminary machine-learning model with indirect calorimetry.

Dear Dr. Jaafar and Reviewer #2,

We thank the academic editor and reviewer #2 for their thorough and constructive evaluation. We have revised the manuscript to address all the points raised. All changes made in this revision are highlighted in yellow in the revised manuscript: “Manuscript_R4_corrected_highlighted.” We respond to each comment below (the reviewer’s text appears in italics).

Reviewer #2 - Major Comments

Reviewer comment. Possible inconsistency in the submitted file: the reviewer PDF appeared to contain a response section referring to a different manuscript on football analytics, PCA/SHAP, and offensive/defensive player profiling.

Response. We apologize for this compilation error. We have verified that both the response letter and the manuscript now correspond exclusively to the present study on resting metabolic rate in soccer players; no material relating to any other manuscript is included. We have re-uploaded a single, coherent set of files (Manuscript, Revised manuscript with tracked changes, and this Response to Reviewers).

Reviewer comment. The machine-learning model should remain clearly framed as exploratory; with n = 40 and R² = 0.169 the model must not be presented as validated, generalizable, or ready for applied use.

Response. We agree and have kept the SVR model described consistently as a preliminary, internal-validation, proof-of-concept analysis throughout the Abstract, Methods, Results, the Figure 3 legend, the Discussion and the Conclusions. We explicitly state that the held-out performance "may be optimistically biased", that the low R² (0.169) leaves most between-individual variability unexplained, and that external validation in larger, independent cohorts is required before any practical use. Earlier wording implying stability, generalization, reliability, or readiness for practice has been removed.

Reviewer comment. The validation strategy and prevention of data leakage must be fully transparent (split ratio; whether tuning, preprocessing, outlier detection, scaling and feature engineering were fitted only on training data; whether the held-out set was used only once; acknowledgement of optimistic bias).

Response. The Methods now state explicitly: a single 80/20 train/validation split; hyperparameter tuning by 5-fold cross-validation performed only within the training subset; preprocessing, outlier screening and RobustScaler scaling fitted exclusively on the training data and then applied unchanged to the held-out set; the held-out set used only once for the final internal-validation estimate; and an explicit acknowledgement that, given the small sample, these estimates may be optimistically biased. We also note that the engineered terms (squared body mass and the body mass × stature interaction) are deterministic transformations and therefore cannot leak information.

Reviewer comment. Multicollinearity among predictors must be handled cautiously: report VIF diagnostics or state clearly that the model is for prediction only and no inference is made about individual predictors.

Response. We have done both. The Methods now report the variance inflation factors computed from the data, approximately 3.5 × 10⁵ (body mass), 6.6 × 10⁵ (body surface area), 1.8 × 10⁵ (body mass × stature), 1.1 × 10⁵ (squared body mass) and 1.6 × 10³ (body mass index), versus 1.7 for age, confirming that the predictor set is collinear by construction. We state explicitly that the models are used solely for prediction and that no inference is made regarding the relative importance or independent contribution of any predictor.

Reviewer comment. Indirect calorimetry procedures require sufficient methodological detail (calibration, fasting, prior-exercise restriction, caffeine/stimulant control, environmental conditions, rest period, measurement duration, steady-state criteria, handling of implausible respiratory quotient values).

Response. We thank the reviewer for this important comment regarding methodological detail in indirect calorimetry procedures. Several of the requested elements were already reported in the manuscript under the “Data sources and measurements” section, including an overnight fast of 7–8 h, a minimum of 12 h abstention from exercise, and a 48 h restriction from alcohol, stimulants, food, and dietary supplements (including coffee, tea, chocolate, carbonated beverages, and energy drinks) prior to testing.

In the Resting metabolic rate subsection, we have now ensured that all remaining methodological requirements are explicitly reported in a single location to improve clarity and completeness. Specifically, we confirm device calibration prior to each test, a 5–10 min familiarization period, testing in the supine position, calculation using the Weir equation, a total measurement window of approximately 12–15 min, environmental control (quiet, thermoneutral room), application of steady-state criteria based on the Academy of Nutrition and Dietetics best-practice guidelines, and the screening of respiratory exchange ratio (RER) values within the physiologically plausible range (0.70–1.00).

Reviewer comment. Numerical consistency across text, tables and figures must be ensured, and "best-performing" must be defined consistently.

Response. We have reconciled all numerical statements. Specifically: (i) the limits of agreement for the Hannon equation in Table 3 were corrected (the signs had been inverted; they now read −321.2 to 1073.4, consistent with a positive mean bias of +376.1); (ii) the concordance-coefficient range in the text was corrected to −0.091 to 0.030 to match Table 3 (Lin's CCC for Kim = −0.091); (iii) the statement that the Owen equation showed "the widest limits of agreement" was corrected, since its span (~1,400 kcal·day⁻¹) is comparable to, not wider than, the other equations; the text now reflects that Owen combines the lowest bias with wide, imprecise limits; (iv) the FAO/WHO relative error in Table 2 was harmonized to 36.4% to match the text and Abstract; (v) bias and error values are now reported to one decimal place consistently across the text, Table 3 and Table 4 (e.g., Owen bias 47.8 kcal·day⁻¹); and (vi) the Figure 1 narrative was aligned with the Hedges' g ranking in Table 4. "Best agreement" is defined explicitly by the two relevant criteria, distinguishing the equation with the highest ICC/CCC (Henry) from that with the lowest bias and RMSE (Owen), while noting that agreement remained poor for all equations.

Reviewer comment. The ethics and consent timeline must be clarified (2014 data collection versus a later ethics-approval code), and informed/parental consent for public sharing of anonymized data must be confirmed.

Response. The Ethics statement clarifies that data were collected during the 2014 Opening Tournament and that the institutional committee (Code CEI-062020-01) granted approval for the retrospective analysis and for the publication of anonymized data. The statement confirms that all participants provided written informed consent, including parental consent for participants under 18 years of age, covering both the research use and the public sharing of anonymized data.

Minor Comments

Reviewer comment. The conclusion that traditional equations are "not adequate" may be too categorical; "showed poor agreement in this sample" would be preferable.

Response. We have softened the Abstract conclusion to "Traditional equations showed poor agreement with indirect calorimetry in this sample of soccer players", and the Discussion/Conclusions consistently frame the limitation as poor agreement and limited external validity rather than categorical inadequacy.

Reviewer comment. Units should be standardized (kcal/day vs kcal·day⁻¹).

Response. The manuscript, figures & tables now uses kcal·day⁻¹ throughout, including the Table 5 column headers (MAE and RMSE) and the corresponding sentence, which previously used bare "kcal". We also standardized the oxygen-consumption notation to VO₂.

Reviewer comment. The Abstract should retain the cautious description of the SVR model and avoid implying external validity.

Response. The Abstract describes the model as a preliminary machine-learning approach and proof of concept, reports the low R² (0.169), and states that predictive capacity remains limited and that external validation is required. No claim of external validity is made.

Reviewer comment. The competing-interests statement should identify any relationship with the indirect-calorimetry device/company, the authors involved, the company role, and who performed the statistical analyses.

Response. The Competing Interests statement identifies the relationship with Breezing Co., names the authors involved (C.A.H.-A. and C.O.R.-G.), describes the nature of the relationship (technical consulting on the indirect-calorimetry methodology) and the honoraria received, states that the company had no role in study design, data collection, analysis, interpretation or the decision to publish, and confirms that the statistical analyses were performed independently by R.Y.-S.

Reviewer comment. The Data Availability Statement should provide a clear, accessible link and specify whether the shared dataset is raw, processed, or anonymized.

Response. The Data Availability Statement provides the Figshare DOI link and now specifies that the deposited dataset is the anonymized, processed, individual-level dataset containing all data needed to reproduce the results.

We believe these revisions address all the points raised and we remain at your disposal for any further clarification.

Sincerely,

The Authors

Attachment Submitted filename: Response_to_Reviewers_R3.docx

# Peer review: Decision Letter 3

**DOI:** 10.1371/journal.pone.0354973.r007

**Authors:** Zulkarnain Jaafar

15 Jul 2026

Estimation of Resting Metabolic Rate in Professional Soccer Players: A Cross-Sectional Study Comparing Traditional Predictive Equations and a Preliminary Machine Learning Model Against Indirect Calorimetry

PONE-D-26-02771R3

Dear Dr. López-Gil,

We’re pleased to inform you that your manuscript has been judged scientifically suitable for publication and will be formally accepted for publication once it meets all outstanding technical requirements.

Within one week, you’ll receive an e-mail detailing the required amendments. When these have been addressed, you’ll receive a formal acceptance letter and your manuscript will be scheduled for publication.

An invoice will be generated when your article is formally accepted. Please note, if your institution has a publishing partnership with PLOS and your article meets the relevant criteria, all or part of your publication costs will be covered. Please make sure your user information is up-to-date by logging into Editorial Manager at Editorial Manager® and clicking the ‘Update My Information' link at the top of the page. For questions related to billing, please contact billing support.

If your institution or institutions have a press office, please notify them about your upcoming paper to help maximize its impact. If they’ll be preparing press materials, please inform our press team as soon as possible -- no later than 48 hours after receiving the formal acceptance. Your manuscript will remain under strict press embargo until 2 pm Eastern Time on the date of publication. For more information, please contact onepress@plos.org.

Kind regards,

Zulkarnain Jaafar

Academic Editor

PLOS One

Additional Editor Comments (optional):

Reviewers' comments:

Reviewer's Responses to Questions

**Comments to the Author**

1. If the authors have adequately addressed your comments raised in a previous round of review and you feel that this manuscript is now acceptable for publication, you may indicate that here to bypass the “Comments to the Author” section, enter your conflict of interest statement in the “Confidential to Editor” section, and submit your "Accept" recommendation.

Reviewer #2: All comments have been addressed

**********

2. Is the manuscript technically sound, and do the data support the conclusions?

The manuscript must describe a technically sound piece of scientific research with data that supports the conclusions. Experiments must have been conducted rigorously, with appropriate controls, replication, and sample sizes. The conclusions must be drawn appropriately based on the data presented.

Reviewer #2: Yes

**********

3. Has the statistical analysis been performed appropriately and rigorously?

Reviewer #2: Yes

**********

4. Have the authors made all data underlying the findings in their manuscript fully available?

The PLOS Data policy requires authors to make all data underlying the findings described in their manuscript fully available without restriction, with rare exception (please refer to the Data Availability Statement in the manuscript PDF file). The data should be provided as part of the manuscript or its supporting information, or deposited to a public repository. For example, in addition to summary statistics, the data points behind means, medians and variance measures should be available. If there are restrictions on publicly sharing data—e.g. participant privacy or use of data from a third party—those must be specified.

Reviewer #2: Yes

**********

5. Is the manuscript presented in an intelligible fashion and written in standard English?

PLOS ONE does not copyedit accepted manuscripts, so the language in submitted articles must be clear, correct, and unambiguous. Any typographical or grammatical errors should be corrected at revision, so please note any specific errors here.

Reviewer #2: Yes

**********

6. Review Comments to the Author

Please use the space provided to explain your answers to the questions above. You may also include additional comments for the author, including concerns about dual publication, research ethics, or publication ethics. (Please upload your review as an attachment if it exceeds 20,000 characters)

Reviewer #2: I thank the authors for their careful and comprehensive responses to my comments. The revised manuscript appears to have addressed the main methodological, interpretive, numerical, ethical, and reporting concerns raised in the previous round. I particularly appreciate the more cautious framing of the machine-learning analysis, the clearer description of the validation procedure, and the improved reporting of the indirect calorimetry protocol.

I have no further substantive concerns and recommend acceptance.

**********

7. PLOS authors have the option to publish the peer review history of their article (what does this mean?). If published, this will include your full peer review and any attached files.

If you choose “no”, your identity will remain anonymous but your review may still be made public.

**Do you want your identity to be public for this peer review?**  For information about this choice, including consent withdrawal, please see our Privacy Policy.

Reviewer #2: No

**********

# Editor report: Acceptance letter

**DOI:** 10.1371/journal.pone.0354973.r008

**Authors:** Zulkarnain Jaafar

PONE-D-26-02771R3

PLOS One

Dear Dr. López-Gil,

I'm pleased to inform you that your manuscript has been deemed suitable for publication in PLOS One. Congratulations! Your manuscript is now being handed over to our production team.

At this stage, our production department will prepare your paper for publication. This includes ensuring the following:

* All references, tables, and figures are properly cited

* All relevant supporting information is included in the manuscript submission,

* There are no issues that prevent the paper from being properly typeset

You will receive further instructions from the production team, including instructions on how to review your proof when it is ready. Please keep in mind that we are working through a large volume of accepted articles, so please give us a few days to review your paper and let you know the next and final steps.

Lastly, if your institution or institutions have a press office, please let them know about your upcoming paper now to help maximize its impact. If they'll be preparing press materials, please inform our press team within the next 48 hours. Your manuscript will remain under strict press embargo until 2 pm Eastern Time on the date of publication. For more information, please contact onepress@plos.org.

You will receive an invoice from PLOS for your publication fee after your manuscript has reached the completed accept phase. If you receive an email requesting payment before acceptance or for any other service, this may be a phishing scheme. Learn how to identify phishing emails and protect your accounts at https://explore.plos.org/phishing.

If we can help with anything else, please email us at customercare@plos.org.

Thank you for submitting your work to PLOS ONE and supporting open access.

Kind regards,

PLOS ONE Editorial Office Staff

on behalf of

Dr. Zulkarnain Jaafar

Academic Editor

PLOS One
